# A Direct Infusion Probe for Rapid Metabolomics of Low-Volume Samples

**DOI:** 10.1021/acs.analchem.2c02918

**Published:** 2022-09-07

**Authors:** Cátia Marques, Liangwen Liu, Kyle D. Duncan, Ingela Lanekoff

**Affiliations:** † Department of Chemistry—BMC, Uppsala University, 75123 Uppsala, Sweden; ‡ Department of Medical Cell Biology, Uppsala University, 75123 Uppsala, Sweden

## Abstract

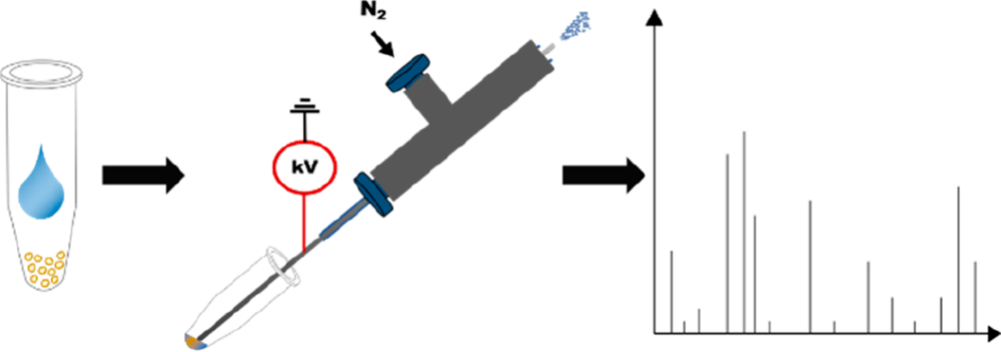

Targeted and nontargeted metabolomics has the potential to evaluate and detect global metabolite changes in biological systems. Direct infusion mass spectrometric analysis enables detection of all ionizable small molecules, thus simultaneously providing information on both metabolites and lipids in chemically complex samples. However, to unravel the heterogeneity of the metabolic status of cells in culture and tissue a low number of cells per sample should be analyzed with high sensitivity, which requires low sample volumes. Here, we present the design and characterization of the direct infusion probe, DIP. The DIP is simple to build and position directly in front of a mass spectrometer for rapid metabolomics of chemically complex biological samples using pneumatically assisted electrospray ionization at 1 μL/min flow rate. The resulting data is acquired in a square wave profile with minimal carryover between samples that enhances throughput and enables several minutes of uniform MS signal from 5 μL sample volumes. The DIP was applied to study the intracellular metabolism of insulin secreting INS-1 cells and the results show that exposure to 20 mM glucose for 15 min significantly alters the abundance of several small metabolites, amino acids, and lipids.

Metabolomics is the comprehensive analysis of small molecules involved in metabolic pathways that drive the biochemistry and function of our cells. Profiling an organism’s dynamic metabolome can lead to objective disease diagnosis,^1,2^ personalized medicine,^3,4^ and realization of fundamental molecular mechanisms involved in complex biological processes.^5,6^ Important applications such as these have catalyzed rapid technology development. For example, there have been significant advancements to achieve higher throughput NMR-based metabolomics, which are intrinsically quantitative with metabolite concentrations proportional to the number of nuclei.^7,8^ However, compared to NMR, the sensitivity and selectivity of mass spectrometry (MS) based metabolomics techniques is imperative for using smaller sample volumes and acquiring a wider metabolite coverage. Thus, MS has evolved into the most universally applied instrumentation for modern metabolomics.

Conventional MS-based metabolomics workflows involve several steps: (i) metabolite extraction and clean up from biofluids, cells, or tissue;^9,10^ (ii) online separation (e.g., liquid chromatography); (iii) and MS detection. Depending on the targeted metabolite compound classes, mass spectrometry can be coupled with either liquid chromatography (LC),^11−14^ gas chromatography (GC),^15,16^ or capillary electrophoresis (CE)^17−19^ for metabolite separation and annotation. While comprehensive separation techniques provide the widest metabolome coverage, achievable sample duty cycles can limit large sample cohort studies and therefore biomedical conclusions.

To increase sample throughput and feasibility for larger cohort studies there is a drive for establishing methods where metabolites extracted from cell and tissue samples are more rapidly profiled and quantified. Higher throughput LC-MS and CE-MS methods have been developed with separation times <2 and 4 min, respectively.^20,21^ Nevertheless, direct infusion MS methods are the fastest reported methods to date, with sample duty cycles of only a few seconds.^22,23^ In addition to increasing sample throughput, there has been considerable effort made to develop direct MS technology for profiling metabolites from volume limited biological samples. For example, ambient ionization techniques such as desorption electrospray ionization (DESI), direct analysis in real time (DART), and liquid microjunction surface sampling probe (LMJ-SSP) have been used to profile metabolites directly from biological surfaces.^24^ Additionally, nanospray based direct infusion MS methods have enabled metabolite profiling in submicroliter volumes.^25−27^

One of the simplest direct MS metabolomics methods reported in the literature is flow injection analysis, with duty cycles <1 min.^28^ Yet, flow injection suffers from longitudinal diffusion from the external pump that limits the number of samples injected online between solvent plugs and causes nonuniform sample elution profiles that limit sensitivity from volume limited injections due to diffusion. To reduce the need for an external pump, eliminate longitudinal diffusion, and instead achieve square wave sample profiles, several probes for direct infusion MS were developed since the late 1990s.^29^ These probes take advantage of the Venturi effect where a flow of gas over the capillary outlet enables direct transport of a sample from its container to the mass spectrometer inlet for subsequent ESI.^29−34^ Depending on the capillary dimensions, which generally have an inner diameter between 100 and 250 μm, flow rates between 5 and 10 μL/min and up to 80 μL/min have been reported. Self-aspirating ESI probes have been constructed both without an applied electrospray voltage^30,31^ and with an applied electrospray voltage placed either in the ESI source^29,33^ or in the sample solution.^32,34^ However, for the analysis of low abundant metabolites the use of voltage for ESI generates higher ionization efficiency and greater metabolite coverage.

Herein, we describe the design, optimization, and characterization of the direct infusion probe (DIP) coupled to MS. The DIP is self-aspirating by using the Venturi effect, and it is made of a stainless-steel capillary that allows for direct application of the high voltage for enhanced ionization. Direct aspiration through the probe for ionization enables analysis of minute sample volumes ≤5 μL and can provide metabolomics of cell or tissue samples down to 20 cells/μL. As a proof-of-principle study, DIP-MS was employed to measure the metabolic response in insulin secreting INS-1 cells after 15 min stimulation with high glucose with simultaneous nontargeted and quantitative targeted mode. The results show significant alterations in several metabolic pathways including both metabolites and lipids. The overall device simplicity makes DIP-MS a new and exciting tool for higher-throughput direct infusion metabolomics.

## Experimental Section

### Chemicals and Prepared Solutions

Solvents for extraction were HLPC-grade methanol (MeOH) (Fisher Scientific), Milli-Q water (H_2_O) from Milli-Q Plus, and formic acid (Merck). For quantification the internal standards oleic acid-d_9_ (FA 18:1-d_9_), LPC 19:0, PC 11:0/11:0, GABA-*d*_2_, Acetylcholine-d_9_, Glucose-d_2_, and Cell Free Amino Acid Mixture-^15^N (Sigma-Aldrich) were prepared in MeOH:H_2_O (9:1) with 0.1% formic acid (Tables S1 and S2). INS-1 cells were exposed to a solution contained 138 mM NaCl, 5.6 mM KCl, 1.2 mM MgCl_2_, 2.6 mM CaCl_2_, and 1, 3, or 20 mM d-glucose at pH 7.4 and room temperature.

### Biological Samples

Frozen intact rat brain tissue (Innovative Research Inc., Novi, MI, USA) was cut into small pieces and added to 35 mL MeOH, generating a metabolite extract of 34.61 mg rat brain tissue per mL. For cell dissociation, the sample was sonicated using an ultrasonic processor (VCX 130, Sonics and Materials, Inc.) for 2 cycles at 50% for a total of 5 min, pulses for 2 s with 1 s pause in between for each cycle. Finally, the sample was centrifuged at 2000 g for 6 min to remove residual tissue debris and the supernatant collected and stored at −20 °C until use.

Immortalized human embryonic kidney 293 cells (HEK293) were cultured in a Dulbecco’s Modified Eagle Medium (DMEM) medium (Gibco) supplemented with 10 v/v % fetal bovine serum (FBS), 1 v/v % nonessential amino acids and 1 v/v % penicillin/streptomycin. Approximately, 1 million cells were collected (Bürker–Türk hemocytometer), washed three times by centrifugation (200 g for 5 min), and resuspension in 1 mL of saline solution at 37 °C. Metabolites from the cell pellet were extracted with 1 mL of MeOH:H_2_O (9:1) plus 0.1% formic acid, sonicated for 20 min, and centrifuged at 13 000 *g* for 20 min to remove cellular debris to eliminate potential clogging of the capillary. Following, a working solution containing 25 000 cells/mL was prepared and further diluted in triplicates to the final cell densities of 500, 400, 300, 200, 100, 50, and 20 cells/μL, and the solvent was evaporated (SpeedVac Vacuum Concentrator, Thermo Fisher Scientific) for 1.5 h. After addition of 5 μL of internal standard solution (Table S1), the samples were stored at −80 °C.

Rat insulinoma cell line INS-1 clone 832/13 cells were maintained in RPMI 1640 (Invitrogen) containing 10 mM glucose and supplemented with 10% fetal bovine serum, streptomycin (100 μg/mL), penicillin (100 μg/mL), Na-pyruvate (1 mM), l-glutamine (2 mM), and 2-mercaptoethanol (50 μM). Approximately 1 million cells were transferred to 24-well for ∼20 h before exposure to 3 mM glucose for 1 h in 37 °C and then either 1 mM glucose or 20 mM glucose for 15 min. Following that, the cells were quickly rinsed three times with saline solution and lysed with 100 μL of MeOH:H_2_O (9:1) with 0.1% formic acid and internal standards (Table S1). Finally, the samples were sonicated and centrifuged as described above and immediately analyzed.

### Mass Spectrometric Parameters

A QExactive Basic (Thermo Fisher Scientific, Bremen, Germany) was used to acquire data in positive and negative modes (for INS-1 cells data) between *m*/*z* 70–1000 using a mass resolution of 140 000 (*m/*Δ*m* at *m*/*z* 200). Data presented was acquired in positive mode unless stated otherwise. Detailed information about the experimental parameters used can be found in Table S3.

### Direct Infusion Probe (DIP)

The DIP probe was constructed using a 7 cm stainless-steel capillary (320:50 μm OD:ID) (MS Ekspert, Gdańsk, Poland), a PEEK sample tee (0.050 in. through hole) (Upchurch Scientific, Oak Harbor, USA) and Teflon sleeves with 1/16-in. OD and 0.5 and 0.75 mm ID (VICI Valco, USA). The sample vial and probe were positioned using a MIM micromanipulator (Quarter Research and Development, Bend, OR). Once the probe position was optimized, it was held in place using an in-house designed and 3D-printed support attached to the mass spectrometer inlet. Nitrogen gas was supplied at ∼5 bar to generate the self-aspirating Venturi pump.

### Data Analysis

The acquired data were analyzed with the software MZmine 2 (parameters used are detailed in Note S1).^35^ Carryover was calculated by taking the intensity of analyte A in scan 10 or 20 of the new sample that does not contain A, and dividing it with the average intensity detected of A in the original sample in percentage (eq 1)
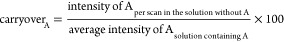
1Other calculations such as, limits of detection (LOD) or concentrations, were based on the integrated signal over 1 min. Metlin and Human metabolome database (HMDB) were used to perform putative assignment of detected peaks via accurate mass (<5 ppm accuracy) for protonated ([M + H]^+^) and sodiated ([M + Na]^+^) adducts. Figures were plotted using Origin 2016 (Thermo Fisher Scientific, Bremen, Germany). Statistical differences for the INS-1 treated cells were evaluated by performing two-tailed unpaired homoscedastic Student’s *t* test. Metabolic pathway networks and Volcano plot for putatively assigned metabolites were obtained through Metaboanalyst.^36^

## Results and Discussion

### Direct Infusion Probe (DIP) Design and Optimization

**Figure 1:**
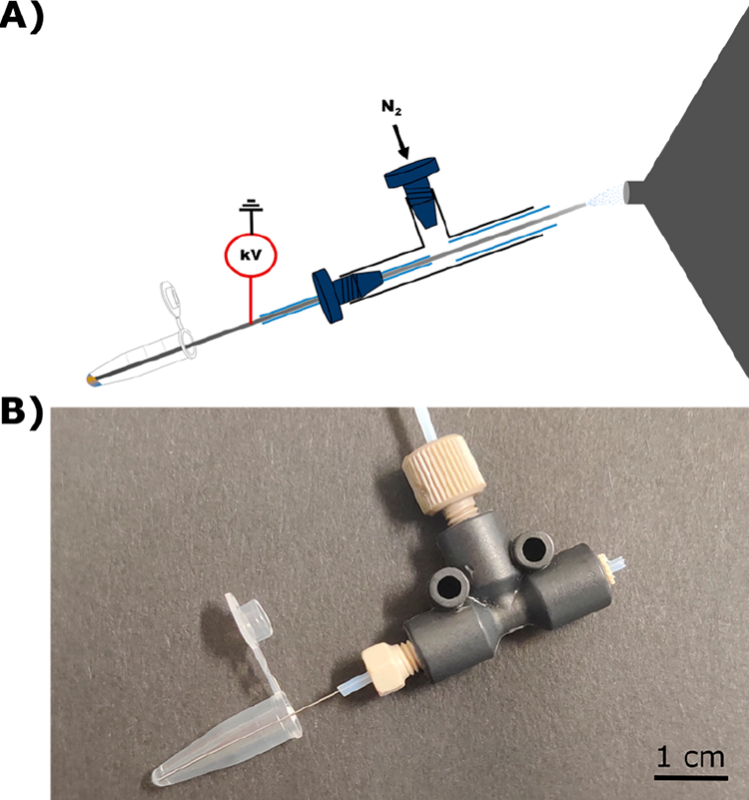
Design of the direct infusion probe (DIP). (A) Schematic overview of the probe in front of the mass spectrometer inlet and (B) photograph of the assembled probe with a 200 μL centrifuge tube.

The DIP was designed to reproducibly sample and ionize minute sample volumes for direct infusion metabolomics. Our design combines application of the high voltage directly on a stainless-steel capillary with a nebulizer constructed from a PEEK sample tee where nitrogen gas is supplied to the top of the tee (Figure 1). The nebulizer allows for self-aspiration through the Venturi self-pumping effect and enhanced solvent desolvation compared to conventional nanoelectrospray sources. Compared to previous miniaturized probes for direct infusion,^32,34^ the DIP has a minimalistic design that incorporates high voltage for efficient ESI directly to flow/ionization capillary. The minimal probe volume of 0.14 μL enables rapid transfer of minute amounts of material directly from the vial to the mass spectrometer inlet for pneumatically assisted electrospray ionization.

For characterization and optimization of the DIP, several key parameters were evaluated using standard solutions. These parameters include probe-to-MS distance, electrospray angle toward the MS inlet, applied nitrogen pressure for self-aspiration, applied electrospray potential, and MS inlet transfer capillary temperature. Good and reproducible data was acquired when the tip of the probe was 2.5 mm away and at a 45° angle directly in front of the mass spectrometer inlet (Figure S1). Furthermore, the optimal conditions were found using a nitrogen pressure of 5 bar (Figure S2), a MS inlet transfer capillary temperature of 300 °C (Figure S3), and an ESI voltage of 1.9 kV (Figure S4). These conditions resulted in a flow rate of 1 μL/min, which was measured by repeated 1 min sampling of solution with known density with weighing steps between each sampling event. The achievable flow rate is attributed to the inner diameter of the stainless-steel capillary in combination with the nitrogen pressure applied to the PEEK tee.^37^ During optimization, it was found that the position in front of the mass spectrometer inlet was important for reproducibility. A holder was therefore 3D-printed to easily position and fix the DIP in front of the mass spectrometer inlet, which minimized accidental movements and maximized reproducible experimental setups. Overall, the DIP probe provides a simple tool for metabolite profiling from very small sample volumes.

### Evaluation of Carryover and Repeatability

**Figure 2:**
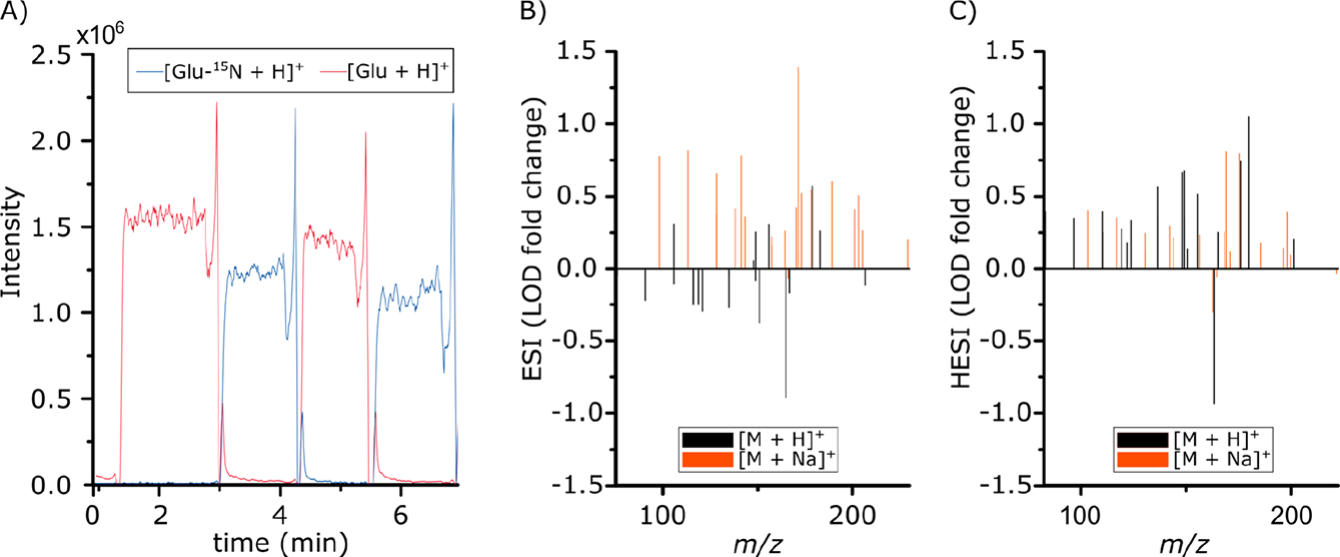
Carryover and comparison between established sources. (A) Extracted ion chronograms of glutamate-^15^N (*m*/*z* 149.0575) in standard solution (blue) and glutamate (*m*/*z* 148.0604) in rat brain extract (red) with a rolling average of 5. (B and C) Fold change of relative LOD for selected metabolite standards between *m*/*z* 75 and 230 plotted for DIP compared to (B) ESI and (C) HESI. Positive values indicated better LOD with the DIP. Protonated adducts are black and sodiated adducts orange.

The DIP has the capacity for rapid sampling of only 5 μL samples and provides square wave sample profiles without longitudinal diffusion. The square wave profile clearly separates samples and simplifies data extraction, analysis, and metabolite annotation. Given that the DIP has a capillary volume of 0.14 μL, the solution within the capillary of the DIP should theoretically be fully exchanged after 8 s with a flow rate of 1 μL/min. The carryover between samples was experimentally determined by switching between standard solutions containing nonendogenous compounds and endogenous compounds from rat brain tissue extract. Thus, either endogenous molecules from the brain extract or standards from the standard solution should be detected. Results for glutamate are depicted in red (endogenous) or blue (^15^N labeled) in the square wave extracted ion chronograms (EIC) in Figure 2A (additional data in Figure S5). Note that the spike at the end of each sampling event is due to a temporarily increased flow of the residual sample inside the capillary.^30^ Despite consecutive sampling, the carryover was considered minimal after 10 scans, which corresponds to ∼8.5 s. At this time, the carryover was 2.5% on average, although it was further decreased to ∼1.1% after 20 scans, ∼17 s (Table S4, eq 1). This shows that the carryover between samples is minimal even without washing steps between, which is similar to other reports.^27^ However, the first 10 scans should not be considered in data analysis due to ongoing sample exchange in the capillary. Furthermore, washing between samples is recommended for high concentration samples or high salt loads.^38^ The rapid exchange of solution in the capillary, minimal dead volume, and minimal carryover between samples has the potential for using the DIP for rapid analysis of low volume samples.

In addition to minimal carryover, high repeatability is desirable for comparative metabolite profiling of minute biological samples. The repeatability of DIP was evaluated by analyzing a standard solution four times within the same day and on three consecutive days to determine intra- and interday repeatabilities, respectively. The intraday RSDs of 24 individual standards as protonated or sodiated species was found to be 4.5% on average, and down to 0.9% at the lowest (Table S5). These data demonstrate that the DIP is a reliable method for direct infusion metabolomics and is comparable with previous reports.^39^ The interday RSD values were higher than the intraday, with 31.2% on average, and 8.3% at the lowest (Table S5). Interday repeatability incorporates the variability introduced by physical day-to-day reassembly of the DIP, causing slight variations in probe positioning and applied gas pressure that ultimately affect flow rate and ionization. We anticipate that variability in ionization is the major component contributing to the larger interday RSD. This is corroborated by the decreased average interday RSD of 20.0% after normalization to the total ion current (Table S5), which compares well with previous reports for commercial ESI sources at 18.8%^39^ and is within the RSD of <20% recommended RSDs from FDA.^40^ Overall, these results demonstrate adequate interday repeatability for the DIP with experimental reassembly, although it is advisable to analyze samples within the same batch using the same probe setup to minimize measurement variations.

### Comparison to Commercially Available ESI Sources

Along with providing measurements with high repeatability, adequate sensitivity is essential for direct infusion metabolomics. The sensitivity of the DIP was compared with commercially available electrospray ionization sources ESI and heated ESI (HESI) using their individual optimized settings and the same mass spectrometer (Table S3) and standard solutions of 25 biologically relevant metabolite and lipid species. Following analysis, 1 min of acquired data with stable TIC signal was extracted for each experiment and the LOD for each standard was calculated according to eq 2, using the slope, *S*, of the calibration curve versus the integrated signal (Table S6) and standard deviation of the linear regression line residuals, σ, (Table S7).^41^
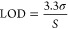
2

The sensitivity was evaluated by the LOD fold change, which was determined by subtracting the LOD obtained with the DIP from the LOD obtained with the respective ESI or HESI source, followed by dividing with the LOD of the DIP. A positive fold change value for a detected standard thereby represents a lower LOD for the DIP, suggesting that a higher sensitivity is afforded to that specific ion. The resulting LOD fold changes for DIP compared to ESI and HESI are plotted in Figure 2B and C, respectively, for standards detected between *m*/*z* 75 and 230 (Table S8). Notably, there are only minor differences in LOD fold-change between the ionization sources, which indicates their functional similarity. However, the fold change is on average 0.21- and 0.28-times better for DIP compared to ESI and HESI, respectively. Furthermore, DIP presents better LODs for 30 and 42 out of 47 detected standard peaks compared with ESI and HESI, respectively. However, the lower flow rate of the DIP (1 μL/min) compared to the ESI and HESI (5 μL/min) generally provides overall longer ion accumulation times and less intense ions per spectra (Table 1). Despite the differences in ion sources, the results show that DIP is fully comparable with the established sources despite its simplicity and low flow rate.

**Table 1:** Ion Accumulation Times (IT) and TIC Values for 20 Consecutive Scans of a Sample of Rat Brain Extract of 0.086 mg/mL Acquired with DIP, ESI, and HESI Sources^a^

	IT (ms) ± RSD (%)	TIC ± RSD (%)
DIP	181.15 ± 3.02	1.17 × 10^7^ ± 3.78
ESI	1.41 ± 8.61	1.54 × 10^9^ ± 7.71
HESI	1.17 ± 6.30	1.84 × 10^9^ ± 5.93

a The flow rate of DIP is 1 μL/min and the flow rate of ESI and HESI is 5 μL/min.

**Figure 3:**
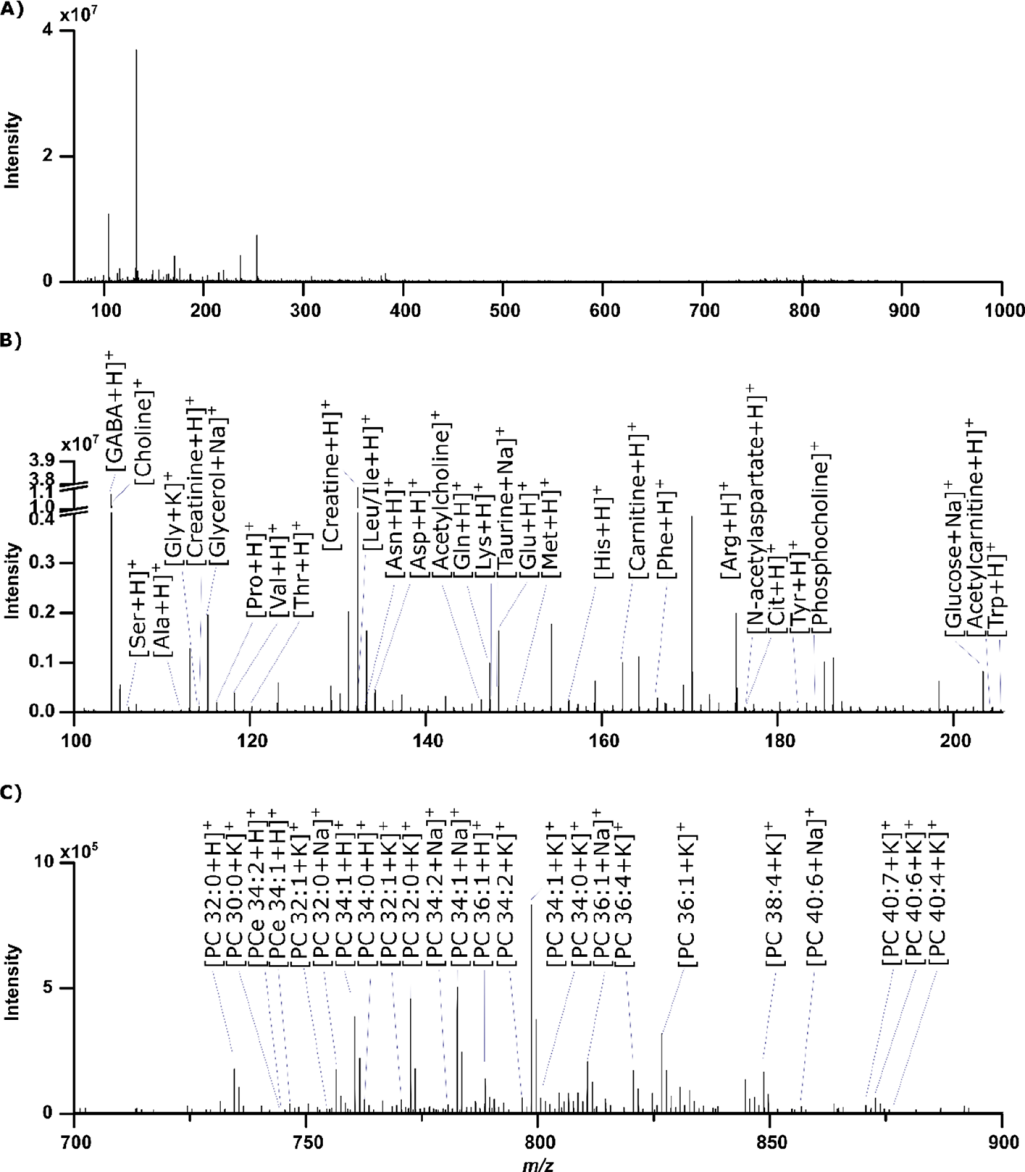
Single scan, without any averaged microscans, high resolution spectrum in positive ion mode from a methanolic rat brain extract analyzed with DIP-MS. (A) Full spectrum from 70 to 1000 Da, (B) same spectrum zoomed into *m/*z 100–206 and with several metabolites annotated, and (C) same spectrum zoomed into *m/*z 700–900 with several annotated lipids. A list of additional putatively annotated peaks is provided in Table S9.

Biological samples are chemically complex and can have a high salt content. The DIP was found to be tolerant to samples with high salt content and to provide a wide range of detected metabolites and lipids in a single scan from an extract of rat brain (Figure 3). The single scan, without any averaged microscans, in Figure 3 corresponds to the extract of 0.8 ng of rat brain tissue, considering analysis of 0.23 nL rat brain extract of 3.46 mg/mL (flow rate of 1 μL/min and duty cycle of ∼14 ms). The mass spectrum consists of a plethora of ions from small polar metabolites to larger hydrophobic lipids, including 19 of the 20 amino acids, neurotransmitters, fatty acids, osmoregulators, sterols, and several phospholipid species, where only a fraction are putatively annotated in Figure 3A–C. A full list of 218 putatively annotated features from which 120 are metabolites in positive ion mode is provided in Table S9. Although, the low flow rate of the DIP overall reduces the number of metabolites detected compared to ESI and HESI, the DIP retains the high diversity of ions detected at one time from a complex sample while only using a minute amount of material.

### Metabolite Detection in Low Cell Number Samples

Most metabolomics studies require tens of microliters of sample and at least one million cells. The DIP was designed to reduce the sample volume and minimize number of cells while retaining a high metabolite coverage. These features are especially important for analysis of a limited number of cells, such as unique samples isolated using fluorescence activated cell sorting (FACS) or primary cells in cultures. To evaluate metabolite detection of samples with low cell densities, 5 μL samples containing methanolic extracts of 20–500 HEK293 cells/μL were analyzed. Selected endogenous molecules were quantified using internal standards according to eq 3, where the endogenous concentration ([endogenous]) was determined from the integrated signal, over 1 min, of the endogenous molecule (Area_endogenous_) to the internal standard (Area_IS_) and multiplied by the concentration of the internal standard ([IS]).
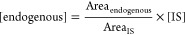
3

**Figure 4:**
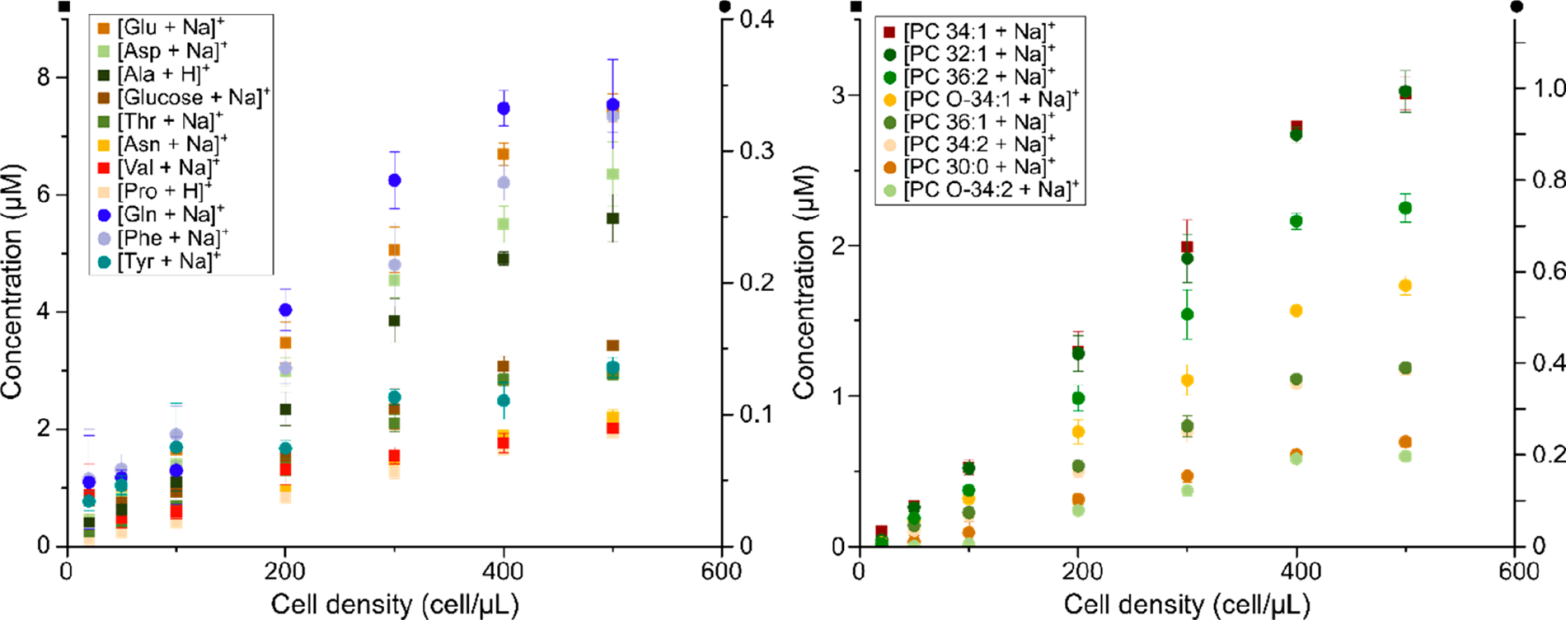
Quantification of endogenous metabolites and lipids in samples with low cell densities. Concentrations of endogenous (A) small metabolites and (B) phospholipids from 5 μL samples with 20 to 500 cells/μL. Note that the *Y*-axes are for species depicted as squares (left) and circles (right). Error bars represent one standard deviation of triplicates of sample preparation. *n* = 3 per cell density, which equals 21 samples in total.

The resulting data show increasing intensities correlating with increasing number of cells/μL. Furthermore, the data suggests that as few as 20 cells/μL generates information from both lipids and metabolites, such as amino acids and glucose, at concentration down to 0.1 μM (Figure 4). The most abundant metabolite of HEK cells quantified here is glutamate followed by aspartic acid and alanine, while phenylalanine has the lowest concentration (Figures 4, S6, and S7). Notably, the most abundant lipid, PC 34:1, has a similar concentration as glucose and the concentration of the second most abundant lipid, PC 32:1, is much lower. Despite the potential to analyze down to 20 cells/μL, it is advisable to use more than 100 cells/μL for a more extensive metabolite profiling. Using an instrument to sort the cells (e.g., FACS) and accurately know the number of cells would be the ideal situation when single cell analysis is required. Nevertheless, the opportunity to use samples with low cell numbers provides new possibilities for isolation and chemical profiling of a small number of unique cells.

### Metabolite Alterations in Glucose Stimulated INS-1 Cells

**Figure 5:**
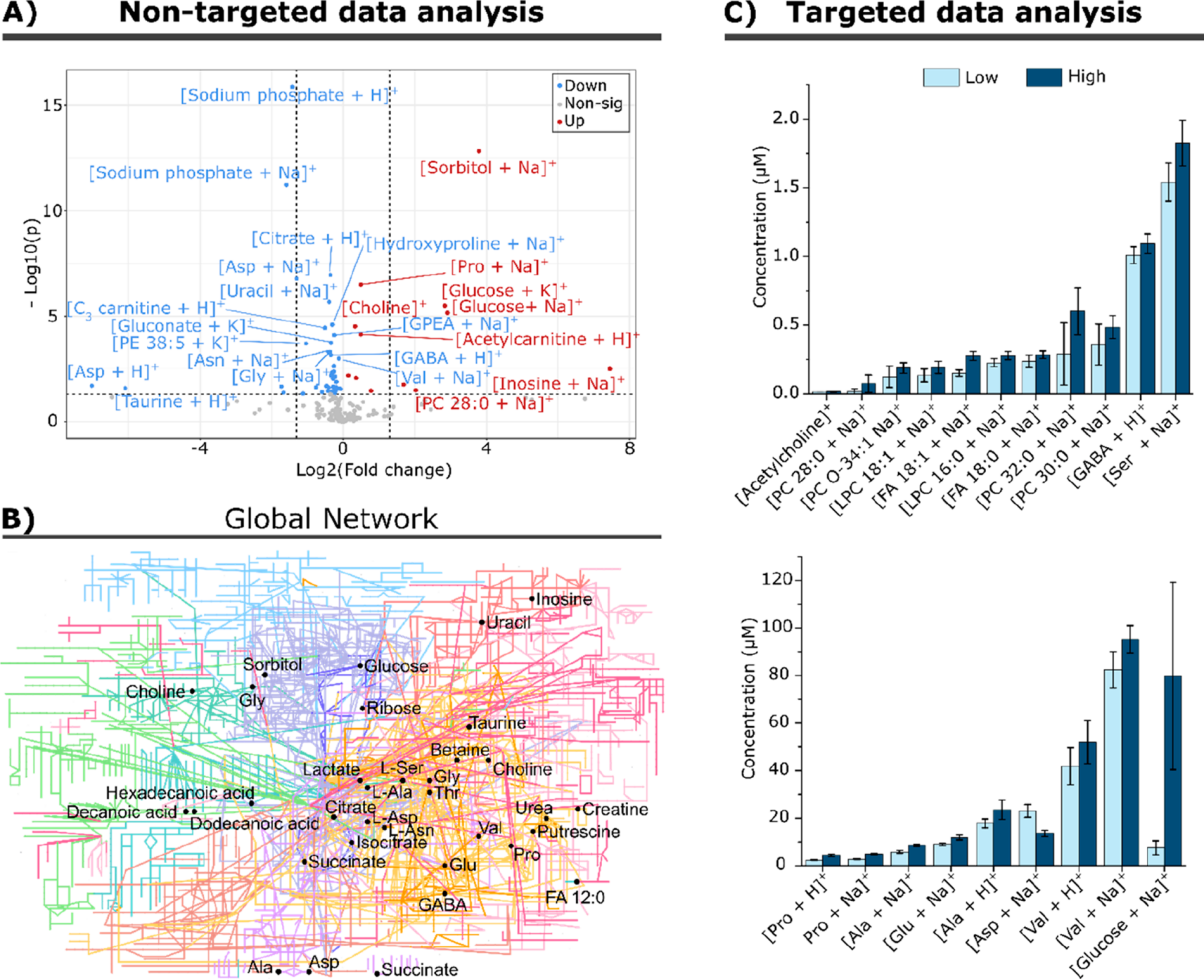
Nontargeted and quantitative targeted metabolite profiling of INS-1 cells exposed to either low (1 mM) or high (20 mM) glucose. (A) Nontargeted data analysis: Volcano plot originated from the global median normalization of a total of 190 features putatively assigned. A total of 56 metabolites were considered significant (*p* < 0.05) according to Student’s *t* test analysis and fold change higher than 1.04. Red and blue correspond to up and down regulated features, respectively, and in gray are the nonsignificant features. (B) Network: The global metabolomic network highlighting in black dots some of the significantly altered metabolites. (C) Targeted data analysis: Concentration of metabolites in INS-1 cell exposed to either low (light blue) or high (dark blue) glucose. All results are significantly different (*p* < 0.05) between the treatment groups according to two-tailed unpaired homoscedastic Student’s *t* test analysis. All results refer to the sodiated adducts of each metabolite, except for valine and proline where the protonated adducts are used. Error bars represent one standard deviation of *n* = 10 of sample preparation from the same batch.

Insulin-secreting INS-1 cells are commonly used to study the release of insulin after exposure to glucose.^42−44^ The DIP was used for a combined nontargeted and quantitative targeted metabolome profiling of cultured INS-1 cells exposed to either 1 or 20 mM of glucose for 15 min. The metabolites were putatively annotated based on accurate mass for nontargeted statistical analysis and the results are presented as a volcano plot in Figure 5A. The results reveal the dynamics of the metabolome and shows significant increases and decreases in numerous metabolites between the two treatment conditions (Table S10). For example, while aspartic acid is 0.4-times decreased in the 20 mM glucose treated cells, sorbitol is only observed in the cells exposed to high glucose environment, which is relevant since glucose is reduced to sorbitol in the polyol pathway. The global metabolic network map in Figure 5B depicts the relation between the significantly altered metabolites, which includes metabolic pathways of amino acid metabolism, in particular alanine, aspartate, and glutamate metabolism and arginine and proline metabolism (Table S11). Additionally, lipid metabolism, including fatty acid biosynthesis and glycerophospholipids metabolism are identified together with the citric acid cycle. These pathways are important to normal cell function and to the regulation of insulin secretion.^45,46^ The nontargeted metabolomics results are in agreement with previously published reports stating that several metabolites are altered in response to high glucose concentrations.^45,46^ In addition to analysis in positive mode, we performed the same experiments in negative mode and the significant metabolite alterations are detected in the similar metabolic pathways (Figure S8 and Tables S12 and S13). Thus, the DIP provides relevant data for exploring intracellular metabolism dynamics from a low number of cells within a short analysis time.

In addition to nontargeted analysis, quantitative results from targeted metabolites were simultaneously acquired with isotopically labeled internal standards to obtain quantitative comparisons. The results show that exposure to 20 mM glucose causes statistically significant (*P* < 0.05) changes in the concentration of wide range of metabolites (Figure 5C). In addition to a higher observed concentration of glucose in the 20 mM glucose treated cells, glutamate, FA 18:1 (oleic acid), and alanine were also increased in cells exposed to high glucose in accordance with previous studies.^47−52^ In particular, glutamate acts as a link between glucose metabolism and insulin release justifying its increase in the presence of high glucose environment.^53^ The increased amount of GABA correlates with the hypothesis that GABA can affect insulin-secretion regulation through the β-cell GABA shunt in response to glucose stimulation.^51,52,54^ Furthermore, since the DIP simultaneously ionizes all material in the cell extract, our data also reveals alterations in PC and LPC species after 15 min of glucose exposure (Figure 5C). Such results have previously only been reported after long-term culture of INS-1 cells in glucotoxic conditions.^55^ These results highlight the capability of the DIP to provide a snapshot with wide molecular coverage of ongoing metabolic events in biological systems. Based on the simplicity of the DIP device in both construction and use, the potential for high throughput analysis, the minimal sample preparation required, and the high metabolite coverage, we anticipate that the DIP will be an important tool for future targeted and nontargeted metabolite profiling of cells and tissue studies where minimal volumes are desired.

## Conclusions

We have presented a probe for direct infusion MS, the DIP, that is simple, robust, and can provide fast analysis of chemically complex biological samples, such as cells and tissue. The probe readily provides minutes of data from samples volumes as low as 5 μL and with a cell density down to 20 cells/μL. This opens up the possibility for new high-throughput metabolomics studies of unique FACS sorted cells, primary cells of limited amounts, or smaller volumes of any type of samples. The DIP is tolerant to high salt loads and chemically complex samples of a range of concentrations and has minimal carryover, thereby reducing the need for laborious and time intensive sample preparation after extraction of metabolites from cells or tissue. Thus, DIP presents itself as a promising alternative to conventional direct infusion mass spectrometry for metabolite profiling. With the potential to combine and simultaneously acquire both nontargeted metabolite profiling and quantitative targeted analysis of selected metabolites, the metabolome can be characterized in both qualitatively and quantitatively. Additionally, the minimal sample preparation and preselection of molecular species simultaneously provides data on a wide range of metabolite and lipid classes. Overall, we anticipate that the minimalist design, method simplicity, and high-quality data generated from minute biological samples makes DIP a valuable tool for the wider metabolomics community.

## Supporting Information Available

Several figures and tables detailing additional experimental conditions, results for evaluation of the performance, quantification, metabolite coverage, details of nontargeted analysis, and suggested altered pathways (PDF)

## References

[ref1] Dutta M.; Singh B.; Joshi M.; Das D.; Subramani E.; Maan M.; Jana S. K.; Sharma U.; Das S.; Dasgupta S.; Ray C. D.; Chakravarty B.; Chaudhury K. Metabolomics Reveals Perturbations in Endometrium and Serum of Minimal and Mild Endometriosis. Sci. Rep. 2018, 8 (1), 1–9. 10.1038/s41598-018-23954-7. 29691425 PMC5915433

[ref2] Yamamoto M.; Shanmuganathan M.; Hart L.; Pai N.; Britz-Mckibbin P. Urinary Metabolites Enable Differential Diagnosis and Therapeutic Monitoring of Pediatric Inflammatory Bowel Disease. Metabolites 2021, 11 (4), 245 10.3390/metabo11040245. 33921143 PMC8071482

[ref3] Coene K. L. M.; Kluijtmans L. A. J.; van der Heeft E.; Engelke U. F. H.; de Boer S.; Hoegen B.; Kwast H. J. T.; van de Vorst M.; Huigen M. C. D. G.; Keularts I. M. L. W.; Schreuder M. F.; van Karnebeek C. D. M.; Wortmann S. B.; de Vries M. C.; Janssen M. C. H.; Gilissen C.; Engel J.; Wevers R. A. Next-Generation Metabolic Screening: Targeted and Untargeted Metabolomics for the Diagnosis of Inborn Errors of Metabolism in Individual Patients. J. Inherited Metab. Dis. 2018, 41 (3), 337–353. 10.1007/s10545-017-0131-6. 29453510 PMC5959972

[ref4] Jacob M.; Lopata A. L.; Dasouki M.; Abdel Rahman A. M. Metabolomics toward Personalized Medicine. Mass Spectrom. Rev. 2019, 38 (3), 221–238. 10.1002/mas.21548. 29073341

[ref5] You X.; Jiang W.; Lu W.; Zhang H.; Yu T.; Tian J.; Wen S.; Garcia-Manero G.; Huang P.; Hu Y. Metabolic Reprogramming and Redox Adaptation in Sorafenib-Resistant Leukemia Cells: Detected by Untargeted Metabolomics and Stable Isotope Tracing Analysis. Cancer Commun. 2019, 39 (1), 17 10.1186/s40880-019-0362-z. PMC6449955 30947742

[ref6] Yurkovich J. T.; Zielinski D. C.; Yang L.; Paglia G.; Rolfsson O.; Sigurjónsson Ó. E.; Broddrick J. T.; Bordbar A.; Wichuk X. K.; Brynjólfsson S.; Palsson S.; Gudmundsson S.; Palsson B. O. Quantitative Time-Course Metabolomics in Human Red Blood Cells Reveal the Temperature Dependence of Human Metabolic Networks. J. Biol. Chem. 2017, 292 (48), 19556–19564. 10.1074/jbc.M117.804914. 29030425 PMC5712598

[ref7] Vignoli A.; Ghini V.; Meoni G.; Licari C.; Takis P. G.; Tenori L.; Turano P.; Luchinat C. High-Throughput Metabolomics by 1D NMR. Angew. Chem., Int. Ed. 2019, 58 (4), 968–994. 10.1002/anie.201804736. PMC6391965 29999221

[ref8] Joseph D.; Sukumaran S.; Chandra K.; Pudakalakatti S. M.; Dubey A.; Singh A.; Atreya H. S. Rapid Nuclear Magnetic Resonance Data Acquisition with Improved Resolution and Sensitivity for High-Throughput Metabolomic Analysis. Magn. Reason. Chem. 2021, 59 (3), 300–314. 10.1002/mrc.5106. 33030750

[ref9] Reyes-Garcés N.; Gionfriddo E. Recent Developments and Applications of Solid Phase Microextraction as a Sample Preparation Approach for Mass-Spectrometry-Based Metabolomics and Lipidomics. TrAC, Trends Anal. Chem. 2019, 113, 172–181. 10.1016/j.trac.2019.01.009.

[ref10] Roca M.; Alcoriza M. I.; Garcia-Cañaveras J. C.; Lahoz A. Reviewing the Metabolome Coverage Provided by LC-MS: Focus on Sample Preparation and Chromatography-A Tutorial. Anal. Chim. Acta 2021, 1147, 38–55. 10.1016/j.aca.2020.12.025. 33485584

[ref11] Zhao S.; Li H.; Han W.; Chan W.; Li L. Metabolomic Coverage of Chemical-Group-Submetabolome Analysis: Group Classification and Four-Channel Chemical Isotope Labeling LC-MS. Anal. Chem. 2019, 91 (18), 12108–12115. 10.1021/acs.analchem.9b03431. 31441644

[ref12] Naz S.; Gallart-Ayala H.; Reinke S. N.; Mathon C.; Blankley R.; Chaleckis R.; Wheelock C. E. Development of a Liquid Chromatography-High Resolution Mass Spectrometry Metabolomics Method with High Specificity for Metabolite Identification Using All Ion Fragmentation Acquisition. Anal. Chem. 2017, 89 (15), 7933–7942. 10.1021/acs.analchem.7b00925. 28641411

[ref13] Pavlaki A.; Begou O.; Deda O.; Farmaki E.; Dotis J.; Gika H.; Taparkou A.; Raikos N.; Papachristou F.; Theodoridis G.; Printza N. Serum-Targeted HILIC-MS Metabolomics-Based Analysis in Infants with Ureteropelvic Junction Obstruction. J. Proteome Res. 2020, 19 (6), 2294–2303. 10.1021/acs.jproteome.9b00855. 32351114

[ref14] Gallart-Ayala H.; Konz I.; Mehl F.; Teav T.; Oikonomidi A.; Peyratout G.; van der Velpen V.; Popp J.; Ivanisevic J. A Global HILIC-MS Approach to Measure Polar Human Cerebrospinal Fluid Metabolome: Exploring Gender-Associated Variation in a Cohort of Elderly Cognitively Healthy Subjects. Anal. Chim. Acta 2018, 1037, 327–337. 10.1016/j.aca.2018.04.002. 30292309

[ref15] Higgins Keppler E. A.; Jenkins C. L.; Davis T. J.; Bean H. D. Advances in the Application of Comprehensive Two-Dimensional Gas Chromatography in Metabolomics. TrAC, Trends Anal. Chem. 2018, 109, 275–286. 10.1016/j.trac.2018.10.015. PMC6333419 30662103

[ref16] Mu Y.; Zhou Y.; Wang Y.; Li W.; Zhou L.; Lu X.; Gao P.; Gao M.; Zhao Y.; Wang Q.; Wang Y.; Xu G. Serum Metabolomics Study of Nonsmoking Female Patients with Non-Small Cell Lung Cancer Using Gas Chromatography-Mass Spectrometry. J. Proteome Res. 2019, 18 (5), 2175–2184. 10.1021/acs.jproteome.9b00069. 30892048

[ref17] Gill B.; Jobst K.; Britz-Mckibbin P. Rapid Screening of Urinary 1-Hydroxypyrene Glucuronide by Multisegment Injection-Capillary Electrophoresis-Tandem Mass Spectrometry: A High-Throughput Method for Biomonitoring of Recent Smoke Exposures. Anal. Chem. 2020, 92 (19), 13558–13564. 10.1021/acs.analchem.0c03212. 32901481

[ref18] Duncan K. D.; Lanekoff I. Spatially Defined Surface Sampling Capillary Electrophoresis Mass Spectrometry. Anal. Chem. 2019, 91 (12), 7819–7827. 10.1021/acs.analchem.9b01516. 31124661

[ref19] Duncan K. D.; Lanekoff I. State-of-the-Art Capillary Electrophoresis Mass Spectrometry Methods for Analyzing the Polar Metabolome. In Advanced Mass Spectrometry-Based Analytical Separation Techniques for Probing the Polar Metabolome; The Royal Society of Chemistry, 2021; Chapter 6, pp 125–164. 10.1039/9781839163524-00125.

[ref20] Liang Q.; Liu H.; Xie L.-x.; Li X.; Zhang A.-H. High-Throughput Metabolomics Enables Biomarker Discovery in Prostate Cancer. RSC Adv. 2017, 7 (5), 2587–2593. 10.1039/C6RA25007F.

[ref21] Guder J. C.; Schramm T.; Sander T.; Link H. Time-Optimized Isotope Ratio LC-MS/MS for High-Throughput Quantification of Primary Metabolites. Anal. Chem. 2017, 89 (3), 1624–1631. 10.1021/acs.analchem.6b03731. 28050903

[ref22] Fuhrer T.; Zamboni N. High-Throughput Discovery Metabolomics. Curr. Opin. Biotechnol. 2015, 31, 73–78. 10.1016/j.copbio.2014.08.006. 25197792

[ref23] Zampieri M.; Szappanos B.; Buchieri M. V.; Trauner A.; Piazza I.; Picotti P.; Gagneux S.; Borrell S.; Gicquel B.; Lelievre J.; Papp B.; Sauer U. High-Throughput Metabolomic Analysis Predicts Mode of Action of Uncharacterized Antimicrobial Compounds. Sci. Transl. Med. 2018, 10 (429), eaal3973 10.1126/scitranslmed.aal3973. 29467300 PMC6544516

[ref24] Clendinen C. S.; Monge M. E.; Fernández F. M. Ambient Mass Spectrometry in Metabolomics. Analyst 2017, 142 (17), 3101–3117. 10.1039/C7AN00700K. 28792022 PMC5729766

[ref25] Southam A. D.; Weber R. J. M.; Engel J.; Jones M. R.; Viant M. R. A Complete Workflow for High-Resolution Spectral-Stitching Nanoelectrospray Direct-Infusion Mass-Spectrometry-Based Metabolomics and Lipidomics. Nat. Protoc. 2017, 12 (2), 310 10.1038/nprot.2016.156. 28079878

[ref26] Li Y.; Bouza M.; Wu C.; Guo H.; Huang D.; Doron G.; Temenoff J. S.; Stecenko A. A.; Wang Z. L.; Fernández F. M. Sub-Nanoliter Metabolomics via Mass Spectrometry to Characterize Volume-Limited Samples. Nat. Commun. 2020, 11 (1), 5625 10.1038/s41467-020-19444-y. 33159052 PMC7648103

[ref27] Wei Z.; Xie Z.; Kuvelkar R.; Shah V.; Bateman K.; McLaren D. G.; Cooks R. G. High-Throughput Bioassays Using “Dip-and-Go” Multiplexed Electrospray Mass Spectrometry. Angew. Chem., Int. Ed. 2019, 58 (49), 17594–17598. 10.1002/anie.201909047. 31589796

[ref28] Sarvin B.; Lagziel S.; Sarvin N.; Mukha D.; Kumar P.; Aizenshtein E.; Shlomi T. Fast and Sensitive Flow-Injection Mass Spectrometry Metabolomics by Analyzing Sample-Specific Ion Distributions. Nat. Commun. 2020, 11 (1), 1–11. 10.1038/s41467-020-17026-6. 32581242 PMC7314751

[ref29] Geromanos S.; Philip J.; Freckleton G.; Tempst P. InJection adaptable fIne Ionization Source (‘JaFIS’) for Continuous Flow Nano-Electrospray. Rapid Commun. Mass Spectrom. 1998, 12 (9), 551–556. 10.1002/(SICI)1097-0231(19980515)12:9<551::AID-RCM198>3.0.CO;2-Y. 9588029

[ref30] Santos V. G.; Regiani T.; Dias F. F. G.; Romão W.; Jara J. L. P.; Klitzke C. F.; Coelho F.; Eberlin M. N. Venturi Easy Ambient Sonic-Spray Ionization. Anal. Chem. 2011, 83 (4), 1375–1380. 10.1021/ac102765z. 21235233

[ref31] Schwab N. v.; Porcari A. M.; Coelho M. B.; Schmidt E. M.; Jara J. L.; Visentainer J. v.; Eberlin M. N. Easy Dual-Mode Ambient Mass Spectrometry with Venturi Self-Pumping, Canned Air, Disposable Parts and Voltage-Free Sonic-Spray Ionization. Analyst 2012, 137 (11), 2537–2540. 10.1039/c2an16312h. 22349120

[ref32] Han J.; Han F.; Ouyang J.; Li Q.; Na N. Venturi-Electrosonic Spray Ionization Cataluminescence Sensor Array for Saccharides Detection. Anal. Chem. 2013, 85 (16), 7738–7744. 10.1021/ac400948k. 23859117

[ref33] Tonin A. P. P.; Poliseli C. B.; Ribeiro M. A. S.; Moraes L. A. B.; Visentainer J. v.; Eberlin M. N.; Meurer E. C. Venturi Electrospray Ionization: Principles and Applications. Int. J. Mass. Spectrom. 2018, 431, 50–55. 10.1016/j.ijms.2018.06.004.

[ref34] Yu Q.; Zhang J.; Ni K.; Qian X.; Wang X. Characterization and Application of a Self-Aspirating Electrospray Source with Pneumatic-Assisted Ionization. J. Mass Spectrom. 2017, 52 (2), 109–115. 10.1002/jms.3907. 28074623

[ref35] Pluskal T.; Castillo S.; Villar-Briones A.; Orešič M. MZmine 2: Modular Framework for Processing, Visualizing, and Analyzing Mass Spectrometry-Based Molecular Profile Data. BMC Bioinf. 2010, 11 (1), 395 10.1186/1471-2105-11-395. PMC2918584 20650010

[ref36] Pang Z.; Zhou G.; Ewald J.; Basu N.; Xia J.; et al. Using MetaboAnalyst 5. 0 for LC – HRMS Spectra Processing, Multi-Omics Integration and Covariate Adjustment of Global Metabolomics Data. Nat. Protoc. 2022, 17, 1735–1761. 10.1038/s41596-022-00710-w. 35715522

[ref37] Schmidt A.; Karas M.; Dülcks T. Effect of Different Solution Flow Rates on Analyte Ion Signals in Nano-ESI MS, or: When Does ESI Turn into Nano-ESI?. J. Am. Soc. Mass. Spectrom. 2003, 14 (5), 492–500. 10.1016/S1044-0305(03)00128-4. 12745218

[ref38] Lin L.; Yu Q.; Yan X.; Hang W.; Zheng J.; Xing J.; Huang B. Direct Infusion Mass Spectrometry or Liquid Chromatography Mass Spectrometry for Human Metabonomics? A Serum Metabonomic Study of Kidney Cancer. Analyst 2010, 135 (11), 2970–2978. 10.1039/c0an00265h. 20856980

[ref39] Kirwan J. A.; Weber R. J. M.; Broadhurst D. I.; Viant M. R. Direct Infusion Mass Spectrometry Metabolomics Dataset: A Benchmark for Data Processing and Quality Control. Sci. Data 2014, 1, 1–13. 10.1038/sdata.2014.12. PMC4381748 25977770

[ref40] Bioanalytical Method Validation; U.S. Food and Drug Administration, 2018.

[ref41] International Conference on Harmonisation. In ICH Topic Q 2 (R1) Validation of Analytical Procedures: Text and Methodologypedia of Toxicology; EMA, 2006.

[ref42] Asfari M.; Janjic D.; Meda P.; Li G.; Halban P. A.; Wollheim C. B. Establishment of 2-Mercaptoethanol-Dependent Differentiated Insulin-Secreting Cell Lines. Endocrinology 1992, 130 (1), 167–178. 10.1210/endo.130.1.1370150. 1370150

[ref43] Ullrich S.; Abel K. B.; Lehr S.; Greger R. Effects of Glucose, Forskolin and Tolbutamide on Membrane Potential and Insulin Secretion in the Insulin Secreting Cell Line INS-1. Pfluegers Arch. 1996, 432 (4), 630–636. 10.1007/s004240050179. 8764963

[ref44] Hohmeier H. E.; Mulder H.; Chen G.; Henkel-Rieger R.; Prentki M.; Newgard C. B. Isolation of INS-1-Derived Cell Lines with Robust ATP-Sensitive K+ Channel-Dependent and -Independent Glucose-Stimulated Insulin Secretion. Diabetes 2000, 49 (3), 424–430. 10.2337/diabetes.49.3.424. 10868964

[ref45] Newsholme P.; Bender K.; Kiely A.; Brennan L. Amino Acid Metabolism, Insulin Secretion and Diabetes. Biochem. Soc. Trans. 2007, 35 (5), 1180–1186. 10.1042/BST0351180. 17956307

[ref46] Spégel P.; Mulder H. Metabolomics Analysis of Nutrient Metabolism in β-Cells. J. Mol. Biol. 2020, 432 (5), 1429–1445. 10.1016/j.jmb.2019.07.020. 31325441

[ref47] Huang M.; Joseph J. W. Metabolomic Analysis of Pancreatic β-Cell Insulin Release in Response to Glucose. Islets 2012, 4 (3), 210–222. 10.4161/isl.20141. 22847496 PMC3442819

[ref48] Huang M.; Joseph J. W. Assessment of the Metabolic Pathways Associated with Glucose-Stimulated Biphasic Insulin Secretion. Endocrinology 2014, 155 (5), 1653–1666. 10.1210/en.2013-1805. 24564396

[ref49] Spégel P.; Sharoyko V. v.; Goehring I.; Danielsson A. P. H.; Malmgren S.; Nagorny C. L. F.; Andersson L. E.; Koeck T.; Sharp G. W. G.; Straub S. G.; Wollheim C. B.; Mulder H. Time-Resolved Metabolomics Analysis of β-Cells Implicates the Pentose Phosphate Pathway in the Control of Insulin Release. Biochem. J. 2013, 450 (3), 595–605. 10.1042/BJ20121349. 23282133

[ref50] Spégel P.; Andersson L. E.; Storm P.; Sharoyko V.; Göhring I.; Rosengren A. H.; Mulder H. Unique and Shared Metabolic Regulation in Clonal β-Cells and Primary Islets Derived from Rat Revealed by Metabolomics Analysis. Endocrinology 2015, 156 (6), 1995–2005. 10.1210/en.2014-1391. 25774549

[ref51] Pizarro-Delgado J.; Braun M.; Hernández-Fisac I.; Martín-Del-Río R.; Tamarit-Rodriguez J. Glucose Promotion of GABA Metabolism Contributes to the Stimulation of Insulin Secretion in β-Cells. Biochem. J. 2010, 431 (3), 381–389. 10.1042/BJ20100714. 20695849

[ref52] Li C.; Liu C.; Nissim I.; Chen J.; Chen P.; Doliba N.; Zhang T.; Nissim I.; Daikhin Y.; Stokes D.; Yudkoff M.; Bennett M. J.; Stanley C. A.; Matschinsky F. M.; Naji A. Regulation of Glucagon Secretion in Normal and Diabetic Human Islets by γ-Hydroxybutyrate and Glycine. J. Biol. Chem. 2013, 288 (6), 3938–3951. 10.1074/jbc.M112.385682. 23266825 PMC3567647

[ref53] Maechler P.; Wollheim C. B. Mitochondrial Glutamate Acts as a Messenger in Glucose-Induced Insulin Exocytosis. Nature 1999, 402 (6762), 685–689. 10.1038/45280. 10604477

[ref54] Sorenson R. L.; Garry D. G.; Brelje T. C. Structural and Functional Considerations of GABA in Islets of Langerhans: β-Cells and Nerves. Diabetes 1991, 40 (11), 1365–1374. 10.2337/diabetes.40.11.1365. 1936599

[ref55] Nyblom H. K.; Nord L. I.; Andersson R.; Kenne L.; Bergsten P. Glucose-Induced de Novo Synthesis of Fatty Acyls Causes Proportional Increases in INS-1E Cellular Lipids. NMR Biomed. 2008, 21 (4), 357–365. 10.1002/nbm.1197. 17691080

