# Supplementary material for: A Direct Infusion Probe for Rapid Metabolomics of Low-Volume Samples
Source: Anal Chem. 2022 Sep 7;94(37):12875–83. doi: 10.1021/acs.analchem.2c02918 (PMC9494293; doi:10.1021/acs.analchem.2c02918)
Supplement: Supplementary file 1 — ac2c02918_si_001.pdf [file ac2c02918_si_001.pdf]

Supporting Information

A direct infusion probe for rapid metabolomics of low-volume samples

Cátia Marques[1], Liangwen Liu [2], Kyle D. Duncan[1], Ingela Lanekoff[1*]

[1] Department of Chemistry – BMC, Uppsala University, 75123 Uppsala, Sweden

[2] Department of Medical Cell Biology, Uppsala University, 75123 Uppsala, Sweden

*Corresponding author:

Prof. Ingela Lanekoff

Ingela.Lanekoff@kemi.uu.se

Dept. of Chemistry-BMC (576)

Uppsala University

751 23 Uppsala

Sweden

Table of Contents

Table S1. 3
Table S2. 4
Table S3. 4
Figure S1. 5
Note S1 5
Figure S2. 6
Figure S3. 6
Figure S4. 7
Figure S5. 7
Table S4. 8
Table S5. 9
Table S6. 10
Table S7. 11
Table S8. 12
Table S9. 13
Figure S6. 17
Figure S7. 18
Table S10. 19
Table S11. 20
Figure S8 20
Table S12. 21
Table S13. 21

Table S1. Concentrations (µM) of standard solutions used in the different experiments.

Concentration (µM)	**optimization**	**Carryover**	**RBE 400-times diluted**	**HEK293 cells**	**RSD DATA**	**INS1 cells**
**LPC 19:0**	5.00	3.57	0.14	1.22	0.83	1.53
**PG 30:0**	2.50	32.13	1.27	0.90	1.12	1.29
**PE 30:0**	2.50	34.46	1.36	0.90	1.33	1.24
**Acetylcholine-d_9**	5.00	1.54	0.06	0.66	0.19	0.90
**Glucose-d_2**	5.00	16.32	0.64	0.75	0.93	0.90
**GABA-d_2**	5.00	9.99	0.39	0.70	0.99	0.13
**PC 11:0/11:0**	-	7.26	0.29	-	0.78	1.17
**FA 18:1-d_9**	-	3.01	0.12	-	0.37	0.48
**Histidine-^{15}N**	1.7	1.51	0.06	0.10	0.08	0.27
**Methionine-^{15}N**	2.7	2.42	0.10	0.15	0.13	0.43
**Tyrosine-^{15}N**	3.3	3.02	0.12	0.19	0.16	0.54
**Arginine-^{15}N**	5.0	4.53	0.18	0.29	0.24	0.81
**Lysine-^{15}N**	5.7	5.14	0.20	0.33	0.28	0.92
**Cysteine-^{15}N**	6.7	6.05	0.24	0.39	0.32	1.08
**Glutamine-^{15}N**	6.7	6.05	0.24	0.39	0.32	1.08
**Phenylalanine-^{15}N**	6.7	6.05	0.24	0.39	0.32	1.08
**Tryptophan-^{15}N**	6.7	6.05	0.24	0.39	0.32	1.08
**Proline-^{15}N**	6.7	6.05	0.24	0.39	0.32	1.08
**Isoleucine-^{15}N**	9.3	8.46	0.33	0.54	0.45	1.51
**Serine-^{15}N**	9.7	8.77	0.35	0.56	0.47	1.56
**Threonine-^{15}N**	11.0	9.97	0.39	0.64	0.53	1.78
**Valine-^{15}N**	12.0	10.88	0.43	0.70	0.58	1.94
**Leucine-^{15}N**	15.0	13.60	0.54	0.87	0.73	2.43
**Glutamic acid-^{15}N**	16.0	14.51	0.57	0.93	0.78	2.59
**Glycine-^{15}N**	19.3	17.53	0.69	1.12	0.94	3.13
**Alanine-^{15}N**	25.0	22.67	0.89	1.45	1.21	4.04
**Aspartic acid-^{15}N**	28.0	25.39	1.00	1.63	1.36	4.53
**Asparagine-^{15}N**	28.0	25.39	1.00	1.63	1.36	4.53

Table S2. Concentrations (µM) of standards used to assess LODs. The values are the average of triplicates of sample preparation.

	IS1	IS2	IS3	IS4	IS5	IS6	IS7	IS8
**LPC 19:0**	0.00	0.12	0.24	0.36	0.01	0.58	0.70	0.82
**PG 30:0**	0.04	1.10	2.16	3.26	0.06	5.20	6.31	7.41
**PE 30:0**	0.04	1.18	2.31	3.50	0.06	5.58	6.76	7.95
**Acetylcholine-d_9**	0.00	0.05	0.10	0.16	0.00	0.25	0.30	0.36
**Glucose-d_2**	0.02	0.56	1.10	1.66	0.03	2.64	3.20	3.77
**GABA-d_2**	0.01	0.34	0.67	1.01	0.02	1.62	1.96	2.30
**PC 11:0/11:0**	0.01	0.25	0.49	0.74	0.01	1.18	1.43	1.68
**FA 18:1-d_9**	0.00	0.10	0.20	0.31	0.01	0.49	0.59	0.69
**Histidine-^{15}N**	0.00	0.05	0.10	0.15	0.00	0.25	0.30	0.35
**Methionine-^{15}N**	0.00	0.08	0.16	0.25	0.01	0.39	0.48	0.56
**Tyrosine-^{15}N**	0.00	0.10	0.20	0.31	0.01	0.49	0.59	0.70
**Arginine-^{15}N**	0.01	0.16	0.30	0.46	0.01	0.73	0.89	1.05
**Lysine-^{15}N**	0.01	0.18	0.35	0.52	0.01	0.83	1.01	1.19
**Cysteine-^{15}N**	0.01	0.21	0.41	0.61	0.01	0.98	1.19	1.40
**Glutamine-^{15}N**	0.01	0.21	0.41	0.61	0.01	0.98	1.19	1.40
**Phenylalanine-^{15}N**	0.01	0.21	0.41	0.61	0.01	0.98	1.19	1.40
**Tryptophan-^{15}N**	0.01	0.21	0.41	0.61	0.01	0.98	1.19	1.40
**Proline-^{15}N**	0.01	0.21	0.41	0.61	0.01	0.98	1.19	1.40
**Isoleucine-^{15}N**	0.01	0.29	0.57	0.86	0.02	1.37	1.66	1.95
**Serine-^{15}N**	0.01	0.30	0.59	0.89	0.02	1.42	1.72	2.02
**Threonine-^{15}N**	0.01	0.34	0.67	1.01	0.02	1.62	1.96	2.30
**Valine-^{15}N**	0.01	0.37	0.73	1.11	0.02	1.76	2.14	2.51
**Leucine-^{15}N**	0.02	0.46	0.91	1.38	0.03	2.20	2.67	3.14
**Glutamate-^{15}N**	0.02	0.50	0.97	1.47	0.03	2.35	2.85	3.35
**Glycine-^{15}N**	0.02	0.60	1.18	1.78	0.03	2.84	3.44	4.05
**Alanine-^{15}N**	0.03	0.77	1.52	2.30	0.04	3.67	4.45	5.23
**Aspartic acid-^{15}N**	0.03	0.87	1.70	2.58	0.05	4.11	4.98	5.86
**Asparagine-^{15}N**	0.03	0.87	1.70	2.58	0.05	4.11	4.98	5.86

Table S3. Summary of mass spectrometry parameters used for the different sources.

	DIP	ESI	HESI
**Flow rate (µL/min)**	1	5	5
**Voltage (kV)**	+1.9 / -1.6	3.2	3.2
**N_2 pressure (bar)**	5	-	-
**Capillary Temperature (°C)**	300	325	325
**Sheath Gas**	-	3	3
**Auxiliary Gas**	-	7	5
**Auxiliary Gas temperature (°C)**	-	-	60
**Maximum injection time, IT (ms)**	300	50	50
**Autogain control**	1×10^6	1×10^6	1×10^6

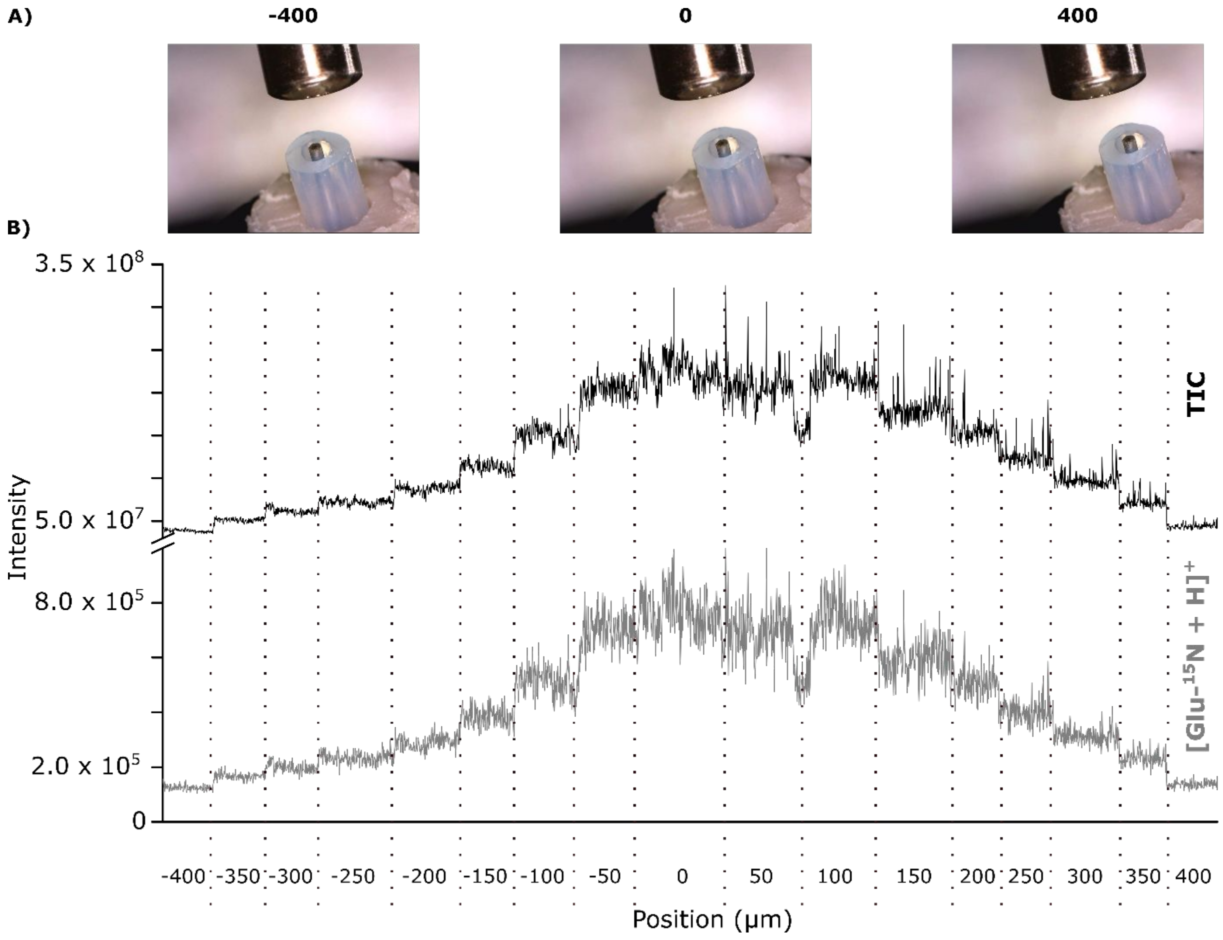

Figure S1. The influence of the DIP positioning relative to the MS inlet was evaluated by moving the DIP in steps of 50 µm from left to right. The position at 0 µm represents the alignment of the probe with the center of the inlet, negative values represent motion towards the left side and the positive values motion towards the right side. A) Images from alignment at -400, 0 and 400 µm. B) TIC and $[Glu\text{-}^{15}N + H]^+$ intensity variation depending on probe position in front of the mass spectrometer.

Note S1. Parameters used with MZmine2.

Raw files were imported to MSmine 2.53. Mass detection was performed for all files using the scans over 1 min of data and using noise level of 1E3. The building of the chromatograms was done over the whole time of each file with a minimum time span of 0.01 min, minimum height of 1E3, and *m/z* tolerance of 0.0002 (or 5 ppm). Peak alignment was performed using the Join alignment method where an *m/z* tolerance of 0.0002 (or 5ppm) was considered, the weight for m/z was 100, the retention time tolerance was 100 min and the weight for retention time was 0. The peak identification was performed with a Custom database search using a file containing identification and theoretical m/z for different analytes and respective adducts, considering a m/z tolerance of 0.0002 or 5 ppm and retention time tolerance of 100 min. The results were exported and further analysed in excel.

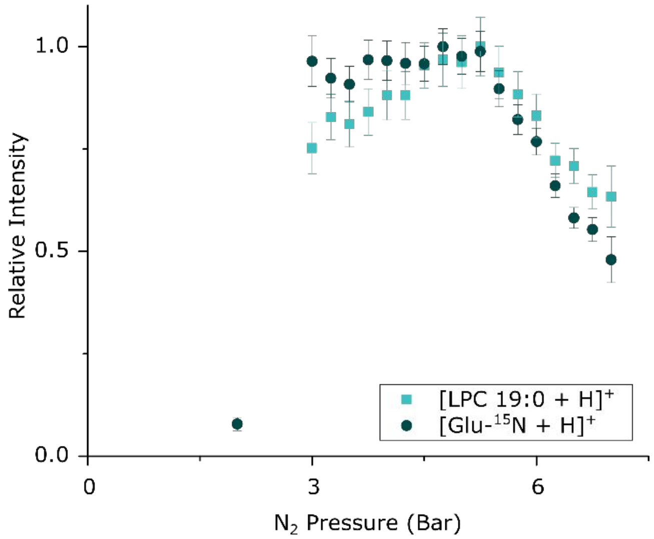

Figure S2. The effect of N_2 pressure on the relative intensity of protonated adducts of ^{15}N-glutamate (circle) and LPC 19:0 (rectangle). The detected intensities are normalized to the highest intensity of each group, specifically, glutamate-^{15}N signal at 4.75 Bar and LPC 19:0 at 5.25 Bar. Error bars depict one standard deviation of scan-to-scan extracted ion chronograms over 1 minute of signal. Standard deviation values are also normalized to the highest intensity of each group.

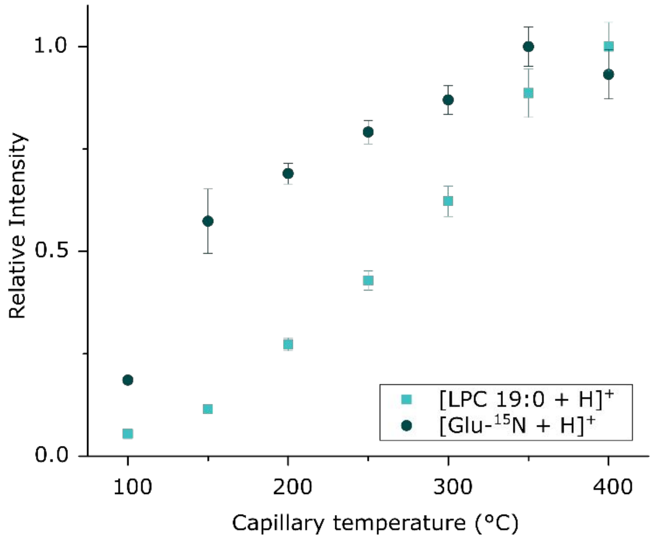

Figure S3. Capillary temperature effect on the signal intensity of protonated adducts of glutamate-^{15}N (circle) and LPC 19:0 (rectangle). Detected intensities are normalized to the highest intensity of each group, specifically, glutamate-^{15}N signal at 350 °C and LPC 19:0 at 400 °C. Error bars depict the standard deviation of scan-to-scan extracted ion chronograms over 1 minute of signal. Standard deviation values are also normalized to the highest intensity of each group.

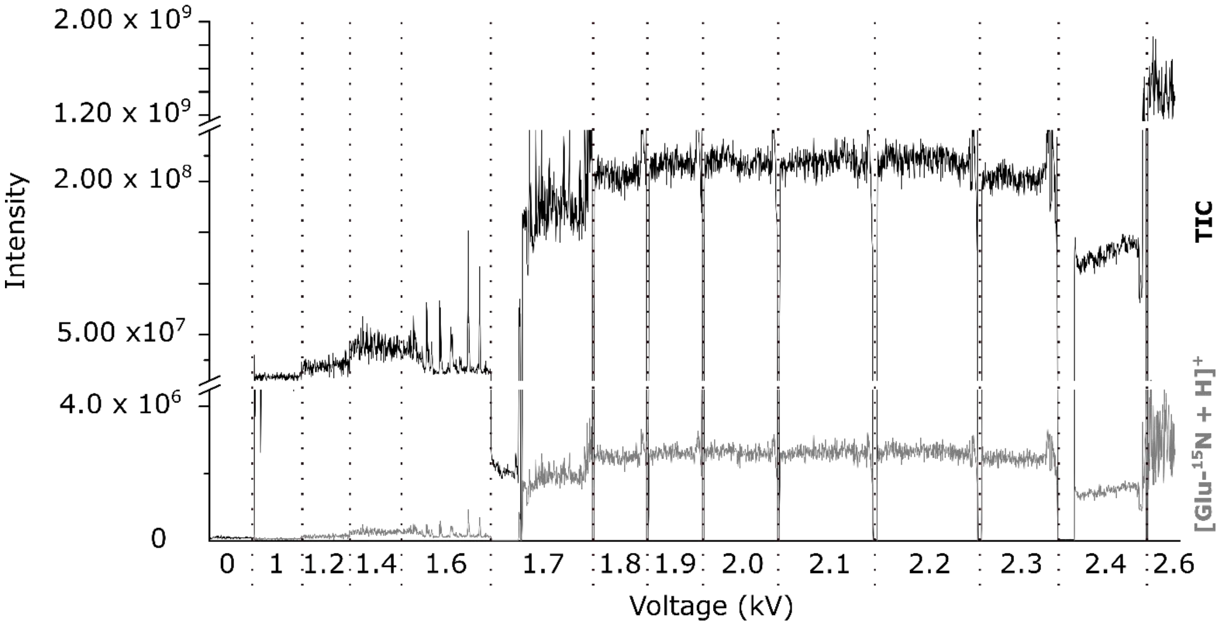

Figure S4. Total ion current (TIC) (black) and extracted ion chronogram of protonated glutamate-^{15}N (grey) at different voltages (kV) applied to the DIP stainless-steal capillary.

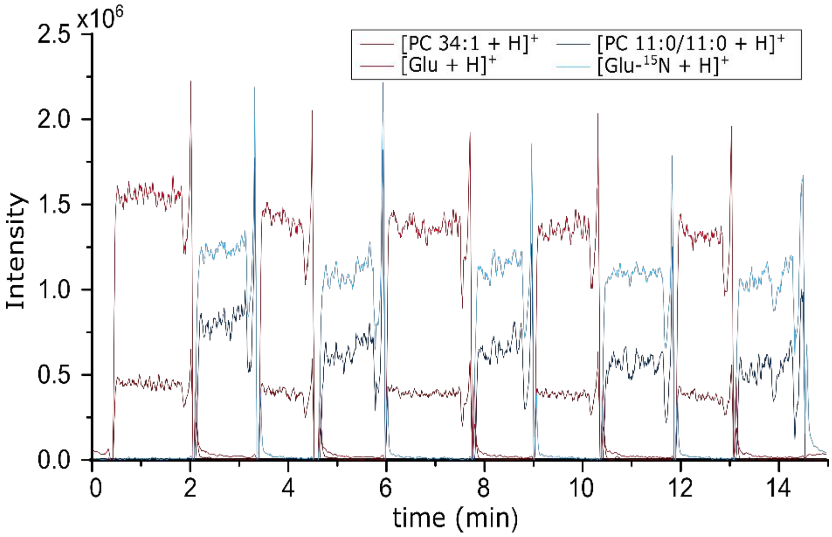

Figure S5. DIP-MS in real time of alternating measurements of rat brain extract and standard solution showing the overlaid rolling average of 5 points of the extracted ion chronograms of protonated endogenous molecules PC 34:1 (*m/z* 760.5851) and glutamate (*m/z* 148.0604) in red and the corresponding protonated standards PC 11:0/11:0 (*m/z* 594.4129) and glutamate-^{15}N (*m/z* 149.0575) in blue.

Table S4. Carryover values for 10 and 20 scans after switching samples for different endogenous metabolites and standards of two alternated mixtures, respectively.

		Carryover	
		**Scan no. 10 (%)**	**Scan no. 20 (%)**
Rat brain extract 10-times diluted	$[PC\ 34{:}1 + H]^+$	9.6	2.1
	$[Glu + H]^+$	5.0	3.4
	$[Glucose + Na]^+$	0.2	0.2
	$[FA\ 20{:}4 + Na]^+$	0.0	0.0
IS standard mixture	$[PC\ 11{:}0/11{:}0 + H]^+$	0.0	0.0
	$[Glu\text{-}^{15}N + H]^+$	3.9	2.6
	$[Glucose\text{-}d_2 + Na]^+$	1.2	0.8
	$[FA\text{-}d_9\ 18{:}1 + Na]^+$	0.0	0.0
	Average	2.5	1.1

Table S5. Intra and interday repeatabilities for DIP-MS analysis. The RSD values of intraday analysis were attained from 4 measurements of the same sample within the same day, while interday analysis considers the analysis of the same sample in 3 consecutive days where the DIP was reassembled each day.

	**RSD for raw areas (%)**		**RSD for TIC area correction (%)**	
	**Intraday**	**Interday**	**Intraday**	**Interday**
**TIC area**	5.1	38.2		
**[Acetylcholine-d_9]$^+$**	2.0	19.5	3.7	18.4
**[Ala-^{15}N + H]$^+$**	1.2	25.2	5.6	16.1
**[Ala-^{15}N + Na]$^+$**	3.9	43.7	7.9	21.4
**[Arg-^{15}N + Na]$^+$**	6.9	32.0	11.3	9.7
**[Asn-^{15}N + H]$^+$**	2.0	21.9	6.7	31.4
**[Asn-^{15}N + Na]$^+$**	3.2	29.6	7.9	11.3
**[Asp-^{15}N + H]$^+$**	1.4	19.8	5.3	30.5
**[Asp-^{15}N + Na]$^+$**	3.6	22.0	8.1	15.0
**[FA 18:1-d_9 + H]$^+$**	9.5	24.2	13.5	18.0
**[FA 18:1-d_9 + Na]$^+$**	10.8	41.3	15.3	27.8
**[GABA-d_2 + H]$^+$**	2.0	31.1	6.2	6.1
**[GABA-d_2 + Na]$^+$**	9.1	50.1	13.6	32.5
**[Gln-^{15}N + H]$^+$**	1.6	20.5	5.8	33.5
**[Gln-^{15}N + Na]$^+$**	4.0	31.3	8.6	11.6
**[Glu-^{15}N + H]$^+$**	2.6	20.9	7.5	31.4
**[Glu-^{15}N + Na]$^+$**	4.0	29.2	8.6	12.0
**[Glucose-d_2 + Na]$^+$**	8.8	32.9	13.2	15.7
**[Gly-^{15}N + H]$^+$**	1.1	21.9	5.4	25.3
**[Gly-^{15}N + Na]$^+$**	4.2	38.3	8.6	15.3
**[Leu/Ile-^{15}N + H]$^+$**	1.5	28.6	5.8	9.6
**[Leu/Ile-^{15}N + Na]$^+$**	4.1	50.9	8.3	27.2
**[LPC 19:0 + H]$^+$**	6.3	36.1	9.9	11.0
**[LPC 19:0 + Na]$^+$**	11.4	37.9	15.9	26.1
**[Met-^{15}N + H]$^+$**	1.5	24.2	5.6	15.2
**[Met-^{15}N + Na]$^+$**	3.5	44.5	7.5	23.4
**[PC 11:0/11:0 + H]$^+$**	5.5	32.1	8.8	13.4
**[PC 11:0/11:0 + Na]$^+$**	8.7	38.3	13.2	26.1
**[PE 30:0 + Na]$^+$**	14.3	36.1	18.7	31.6
**[PG 30:0 + Na]$^+$**	9.2	8.3	13.9	24.5
**[Phe-^{15}N + H]$^+$**	1.8	24.2	6.4	15.3
**[Phe-^{15}N + Na]$^+$**	4.4	45.3	8.7	24.3
**[Pro-^{15}N + H]$^+$**	0.9	23.3	5.4	14.8
**[Pro-^{15}N + Na]$^+$**	4.0	45.3	8.3	21.8
**[Ser-^{15}N + H]$^+$**	1.7	23.0	6.0	27.3
**[Ser-^{15}N + Na]$^+$**	4.9	32.3	9.6	13.0
**[Thr-^{15}N + H]$^+$**	2.1	22.0	7.0	26.0
**[Thr-^{15}N + Na]$^+$**	3.1	33.7	7.6	14.3
**[Trp-^{15}N + H]$^+$**	4.0	23.7	8.5	14.8
**[Trp-^{15}N + Na]$^+$**	7.0	41.6	11.4	21.3
**[Tyr-^{15}N + H]$^+$**	2.7	22.4	7.4	20.6
**[Tyr-^{15}N + Na]$^+$**	3.7	37.1	7.9	17.4
**[Val-^{15}N + H]$^+$**	1.1	26.7	5.3	11.0
**[Val-^{15}N + Na]$^+$**	3.7	47.9	8.1	25.7
Average	4.5	31.2	8.8	20.0

Table S6. Parameters of calibration curves used to determine LOD of DIP, ESI and HESI. In the general trendline of y = S x + b, S is the slope of the trendline, and b is the y-intercept.

	DIP			ESI			HESI		
	S	b	R^2	S	b	R^2	S	b	R^2
**[Acetylcholine-d$_9$]$^+$**	1.8E8	-1.1E6	0.9312	1.7E10	-3.1E8	0.9093	2.4E10	-4.6E8	0.9202
**[Ala-^{15}N + H]$^+$**	2.9E6	8.1E5	0.9546	6.4E8	1.1E8	0.9721	7.3E8	1.0E8	0.9202
**[Ala-^{15}N + Na]$^+$**	4.4E5	1.8E5	0.8788	2.9E7	2.6E7	0.7175	3.6E7	2.6E7	0.8205
**[Arg-^{15}N + H]$^+$**	8.3E6	-9.7E5	0.9471	3.0E9	-3.8E8	0.8849	3.7E9	-4.8E8	0.8623
**[Arg-^{15}N + Na]$^+$**	3.6E5	9.6E4	0.8839	7.4E7	4.2E6	0.7922	1.0E8	3.4E6	0.8531
**[Asn-^{15}N + H]$^+$**	2.8E5	5.0E4	0.9595	1.2E8	-2.6E6	0.9625	1.3E8	-5.2E6	0.9058
**[Asn-^{15}N + Na]$^+$**	4.3E5	9.0E4	0.9210	8.8E7	1.4E7	0.9087	1.1E8	7.9E6	0.9060
**[Asp-^{15}N + H]$^+$**	4.6E5	4.1E5	0.9384	2.4E8	6.6E7	0.9665	2.5E8	8.3E7	0.8905
**[Asp-^{15}N + Na]$^+$**	5.6E5	3.0E5	0.9123	9.7E7	7.9E7	0.8807	1.2E8	8.0E7	0.8785
**[FA 18:1-d$_9$ + H]$^+$**	1.5E5	-3.2E3	0.7107	4.0E7	5.5E5	0.9328	3.3E7	-1.4E5	0.6378
**[FA 18:1-d$_9$ + Na]$^+$**	3.2E6	5.7E5	0.8068	2.3E8	7.2E7	0.5951	2.1E8	5.2E7	0.6020
**[GABA-d$_2$ + H]$^+$**	9.0E6	8.2E5	0.9523	1.4E9	1.5E8	0.9207	1.6E9	1.2E8	0.9273
**[GABA-d$_2$ + Na]$^+$**	4.3E5	9.3E4	0.8002	4.4E7	1.2E7	0.6548	6.7E7	1.1E7	0.8032
**[Gln-^{15}N + H]$^+$**	2.1E6	5.6E4	0.9656	6.1E8	-8.5E6	0.9751	6.6E8	-1.5E7	0.9200
**[Gln-^{15}N + Na]$^+$**	1.5E6	8.8E4	0.8791	2.9E8	1.3E7	0.8834	3.7E8	5.3E6	0.8955
**[Glu-^{15}N + H]$^+$**	1.8E6	4.5E5	0.9664	6.5E8	6.0E7	0.9625	7.0E8	6.2E7	0.9118
**[Glu-^{15}N + Na]$^+$**	1.0E6	2.2E5	0.9148	1.7E8	6.0E7	0.8490	2.1E8	5.9E7	0.8781
**[Glucose-d$_2$ + Na]$^+$**	1.7E6	5.8E5	0.8820	3.7E8	2.4E8	0.7661	4.7E8	2.8E8	0.7928
**[Gly-^{15}N + H]$^+$**	3.9E5	8.9E4	0.9337	1.3E8	6.0E6	0.9530	1.3E8	5.1E6	0.9036
**[Gly-^{15}N + Na]$^+$**	1.8E5	9.4E4	0.8921	1.4E7	1.1E7	0.7728	1.5E7	1.0E7	0.8450
**[His-^{15}N + H]$^+$**	3.0E6	-8.6E4	0.8847	1.0E9	-3.6E7	0.8262	1.2E9	-4.6E7	0.7798
**[His-^{15}N + Na]$^+$**	6.1E5	-3.2E4	0.8367	1.1E8	-3.5E6	0.8110	1.5E8	-4.8E6	0.7716
**[LPC 19:0 + H]$^+$**	1.5E6	-1.4E5	0.8803	1.0E9	-3.7E7	0.9219	1.8E9	-7.7E7	0.8838
**[LPC 19:0 + Na]$^+$**	3.3E6	1.6E5	0.8494	3.0E8	4.5E7	0.8034	4.7E8	4.4E7	0.8878
**[Lys-^{15}N + H]$^+$**	1.9E6	-3.2E5	0.8669	6.4E8	-1.0E8	0.8267	7.2E8	-1.2E8	0.8046
**[Lys-^{15}N + Na]$^+$**	3.0E5	-9.6E4	0.9214	5.4E7	-9.1E6	0.7857	8.2E7	-2.1E7	0.8298
**[Met-^{15}N + H]$^+$**	9.2E6	1.8E5	0.9433	2.5E9	3.2E7	0.9788	2.9E9	3.0E7	0.9315
**[Met-^{15}N + Na]$^+$**	2.2E6	1.2E5	0.8368	2.1E8	1.6E7	0.7419	2.8E8	1.7E7	0.8409
**[PC 11:0/11:0 + H]$^+$**	2.1E6	-4.1E5	0.8684	1.6E9	-9.7E7	0.9108	2.9E9	-2.5E8	0.8709
**[PC 11:0/11:0 + Na]$^+$**	3.8E6	3.2E5	0.8568	3.9E8	1.4E8	0.7253	6.2E8	1.4E8	0.8538
**[PE 30:0 + H]$^+$**	9.2E4	1.7E5	0.8690	9.8E7	2.4E7	0.9474	1.7E8	2.8E7	0.9263
**[PE 30:0 + Na]$^+$**	2.7E5	7.5E5	0.7399	2.9E7	1.2E8	0.6402	5.5E7	1.3E8	0.8546
**[PG 30:0 + H]$^+$**	-	-	-	4.2E6	-2.1E6	0.4889	6.4E6	-4.7E6	0.7945
**[PG 30:0 + Na]$^+$**	4.5E4	6.3E4	0.9000	1.9E7	8.6E6	0.9125	3.4E7	3.2E6	0.9101
**[Phe-^{15}N + H]$^+$**	1.4E7	1.1E6	0.9491	4.7E9	1.7E8	0.9647	5.7E9	1.6E8	0.9221
**[Phe-^{15}N + Na]$^+$**	3.6E6	4.0E5	0.8592	3.7E8	7.5E7	0.7155	5.2E8	7.8E7	0.8234
**[Pro-^{15}N + H]$^+$**	1.2E7	6.0E5	0.9407	2.8E9	6.5E7	0.9660	3.5E9	3.0E7	0.9069
**[Pro-^{15}N + Na]$^+$**	5.8E6	6.1E5	0.8073	3.7E8	8.6E7	0.6756	5.0E8	9.0E7	0.8058
**[Ser-^{15}N + H]$^+$**	5.4E5	7.0E4	0.9475	2.3E8	5.3E6	0.9605	2.6E8	2.9E6	0.9075
**[Ser-^{15}N + Na]$^+$**	5.8E5	1.2E5	0.8970	8.8E7	2.0E7	0.8572	1.0E8	2.0E7	0.8792
**[Thr-^{15}N + H]$^+$**	1.3E6	1.6E5	0.9568	5.3E8	2.6E7	0.9783	6.0E8	2.3E7	0.9253
**[Thr-^{15}N + Na]$^+$**	1.2E6	2.0E5	0.8857	1.6E8	3.3E7	0.8449	1.9E8	3.3E7	0.8722
**[Trp-^{15}N + H]$^+$**	6.1E6	7.6E5	0.9479	2.4E9	1.3E8	0.9591	2.9E9	1.1E8	0.9258
**[Trp-^{15}N + Na]$^+$**	1.9E6	4.7E5	0.8101	3.4E8	5.3E7	0.7739	4.5E8	5.5E7	0.8410
**[Tyr-^{15}N + H]$^+$**	4.9E6	2.4E5	0.9816	2.2E9	2.5E7	0.9709	2.5E9	2.5E7	0.9266
**[Tyr-^{15}N + Na]$^+$**	1.6E6	8.5E4	0.8839	2.7E8	1.6E7	0.8609	3.5E8	1.5E7	0.8919
**[Val-^{15}N + H]$^+$**	9.8E6	1.3E6	0.9477	2.3E9	1.8E8	0.9699	2.7E9	1.7E8	0.9285
**[Val-^{15}N + Na]$^+$**	2.4E6	4.0E5	0.8749	1.6E8	6.3E7	0.6873	2.1E8	6.2E7	0.8055

Table S7. Slope of calibration curve, S, and standard deviation of the linear regression line residuals, σ, values used to calculate LOD of DIP, ESI and HESI according to Eq.2.

	**DIP**		**ESI**		**HESI**	
	S	σ	S	σ	S	σ
**[Acetylcholine-d$_9$]$^+$**	1.8E8	5.92E6	1.7E10	6.52E8	2.4E10	8.50E8
**[Ala-^{15}N + H]$^+$**	2.9E6	1.12E6	6.4E8	1.92E8	7.3E8	3.78E8
**[Ala-^{15}N + Na]$^+$**	4.4E5	2.66E5	2.9E7	3.18E7	3.6E7	2.98E7
**[Arg-^{15}N + H]$^+$**	8.3E6	6.74E5	3.0E9	3.80E8	3.7E9	5.27E8
**[Arg-^{15}N + Na]$^+$**	3.6E5	4.59E4	7.4E7	1.35E7	1.0E8	1.53E7
**[Asn-^{15}N + H]$^+$**	2.8E5	1.14E5	1.2E8	4.66E7	1.3E8	8.31E7
**[Asn-^{15}N + Na]$^+$**	4.3E5	2.30E5	8.8E7	5.49E7	1.1E8	6.75E7
**[Asp-^{15}N + H]$^+$**	4.6E5	2.35E5	2.4E8	8.69E7	2.5E8	1.71E8
**[Asp-^{15}N + Na]$^+$**	5.6E5	3.35E5	9.7E7	7.04E7	1.2E8	8.49E7
**[FA 18:1-d$_9$ + H]$^+$**	1.5E5	1.69E4	4.0E7	2.19E6	3.3E7	5.18E6
**[FA 18:1-d$_9$ + Na]$^+$**	3.2E6	3.70E5	2.3E8	4.40E7	2.1E8	3.98E7
**[GABA-d$_2$ + H]$^+$**	9.0E6	1.57E6	1.4E9	3.08E8	1.6E9	3.56E8
**[GABA-d$_2$ + Na]$^+$**	4.3E5	1.47E5	4.4E7	2.50E7	6.7E7	2.56E7
**[Gln-^{15}N + H]$^+$**	2.1E6	1.74E5	6.1E8	4.61E7	6.6E8	9.20E7
**[Gln-^{15}N + Na]$^+$**	1.5E6	2.34E5	2.9E8	5.02E7	3.7E8	5.97E7
**[Glu-^{15}N + H]$^+$**	1.8E6	3.81E5	6.5E8	1.45E8	7.0E8	2.45E8
**[Glu-^{15}N + Na]$^+$**	1.0E6	3.42E5	1.7E8	7.96E7	2.1E8	8.73E7
**[Glucose-d$_2$ + Na]$^+$**	1.7E6	7.70E5	3.7E8	2.58E8	4.7E8	3.09E8
**[Gly-^{15}N + H]$^+$**	3.9E5	1.25E5	1.3E8	3.91E7	1.3E8	5.89E7
**[Gly-^{15}N + Na]$^+$**	1.8E5	7.45E4	1.4E7	1.01E7	1.5E7	8.82E6
**[His-^{15}N + H]$^+$**	3.0E6	1.23E5	1.0E9	5.62E7	1.2E9	7.72E7
**[His-^{15}N + Na]$^+$**	6.1E5	2.18E4	1.1E8	6.23E6	1.5E8	9.58E6
**[LPC 19:0 + H]$^+$**	1.5E6	1.37E5	1.0E9	7.51E7	1.8E9	1.80E8
**[LPC 19:0 + Na]$^+$**	3.3E6	3.70E5	3.0E8	4.11E7	4.7E8	4.64E7
**[Lys-^{15}N + H]$^+$**	1.9E6	2.74E5	6.4E8	1.17E8	7.2E8	1.42E8
**[Lys-^{15}N + Na]$^+$**	3.0E5	2.55E4	5.4E7	1.10E7	8.2E7	1.25E7
**[Met-^{15}N + H]$^+$**	9.2E6	4.10E5	2.5E9	6.99E7	2.9E9	1.50E8
**[Met-^{15}N + Na]$^+$**	2.2E6	1.64E5	2.1E8	2.36E7	2.8E8	2.32E7
**[PC 11:0/11:0 + H]$^+$**	2.1E6	4.10E5	1.6E9	2.78E8	2.9E9	6.28E8
**[PC 11:0/11:0 + Na]$^+$**	3.8E6	8.89E5	3.9E8	1.35E8	6.2E8	1.46E8
**[PE 30:0 + H]$^+$**	9.2E4	8.36E4	9.8E7	6.22E7	1.7E8	1.28E8
**[PE 30:0 + Na]$^+$**	2.7E5	4.29E5	2.9E7	5.83E7	5.5E7	6.09E7
**[PG 30:0 + H]$^+$**	2.0E3	3.66E4	4.2E6	8.11E6	6.4E6	7.14E6
**[PG 30:0 + Na]$^+$**	4.5E4	3.32E4	1.9E7	1.32E7	3.4E7	2.31E7
**[Phe-^{15}N + H]$^+$**	1.4E7	1.53E6	4.7E9	4.20E8	5.7E9	7.77E8
**[Phe-^{15}N + Na]$^+$**	3.6E6	6.72E5	3.7E8	1.11E8	5.2E8	1.13E8
**[Pro-^{15}N + H]$^+$**	1.2E7	1.45E6	2.8E9	2.48E8	3.5E9	5.22E8
**[Pro-^{15}N + Na]$^+$**	5.8E6	1.34E6	3.7E8	1.20E8	5.0E8	1.15E8
**[Ser-^{15}N + H]$^+$**	5.4E5	8.41E4	2.3E8	3.23E7	2.6E8	5.59E7
**[Ser-^{15}N + Na]$^+$**	5.8E5	1.18E5	8.8E7	2.46E7	1.0E8	2.58E7
**[Thr-^{15}N + H]$^+$**	1.3E6	2.12E5	5.3E8	6.13E7	6.0E8	1.33E8
**[Thr-^{15}N + Na]$^+$**	1.2E6	2.99E5	1.6E8	5.27E7	1.9E8	5.68E7
**[Trp-^{15}N + H]$^+$**	6.1E6	6.78E5	2.4E9	2.30E8	2.9E9	3.89E8
**[Trp-^{15}N + Na]$^+$**	1.9E6	4.01E5	3.4E8	8.58E7	4.5E8	9.17E7
**[Tyr-^{15}N + H]$^+$**	4.9E6	1.59E5	2.2E9	8.84E7	2.5E9	1.69E8
**[Tyr-^{15}N + Na]$^+$**	1.6E6	1.23E5	2.7E8	2.53E7	3.5E8	2.83E7
**[Val-^{15}N + H]$^+$**	9.8E6	1.96E6	2.3E9	3.39E8	2.7E9	6.28E8
**[Val-^{15}N + Na]$^+$**	2.4E6	7.64E5	1.6E8	9.02E7	2.1E8	8.60E7

Table S8. Rounded LOD values and respective rounded fold-changes for DIP, ESI and HESI. Positive values for the fold change display a lower LOD for DIP, and negative values a lower LOD for ESI or HESI.

	LOD (µM)			Fold Change	
	**DIP**	**ESI**	**HESI**	**ESI**	**HESI**
**[Acetylcholine-d$_9$]$^+$**	0.11	0.13	0.12	0.16	0.08
**[Ala-^{15}N + H]$^+$**	1.27	0.99	1.72	-0.22	0.35
**[Ala-^{15}N + Na]$^+$**	2.01	3.66	2.73	0.82	0.35
**[Arg-^{15}N + H]$^+$**	0.27	0.42	0.47	0.57	0.74
**[Arg-^{15}N + Na]$^+$**	0.42	0.60	0.48	0.41	0.14
**[Asn-^{15}N + H]$^+$**	1.34	1.29	2.11	-0.04	0.57
**[Asn-^{15}N + Na]$^+$**	1.78	2.07	2.10	0.16	0.18
**[Asp-^{15}N + H]$^+$**	1.67	1.21	2.29	-0.27	0.37
**[Asp-^{15}N + Na]$^+$**	1.96	2.40	2.43	0.22	0.24
**[FA 18:1-d$_9$ + H]$^+$**	0.37	0.18	0.51	-0.51	0.38
**[FA 18:1-d$_9$ + Na]$^+$**	0.38	0.64	0.63	0.69	0.66
**[GABA-d$_2$ + H]$^+$**	0.57	0.75	0.72	0.31	0.25
**[GABA-d$_2$ + Na]$^+$**	1.12	1.86	1.27	0.66	0.13
**[Gln-^{15}N + H]$^+$**	0.27	0.25	0.46	-0.09	0.68
**[Gln-^{15}N + Na]$^+$**	0.51	0.56	0.53	0.12	0.05
**[Glu-^{15}N + H]$^+$**	0.70	0.74	1.16	0.06	0.67
**[Glu-^{15}N + Na]$^+$**	1.10	1.57	1.39	0.43	0.26
**[Glucose-d$_2$ + Na]$^+$**	1.54	2.32	2.15	0.51	0.40
**[Gly-^{15}N + H]$^+$**	1.05	1.00	1.47	-0.05	0.40
**[Gly-^{15}N + Na]$^+$**	1.37	2.44	1.93	0.78	0.41
**[His + H]$^+$**	0.14	0.18	0.21	0.31	0.52
**[His + Na]$^+$**	0.12	0.18	0.21	0.55	0.80
**[LPC 19:0 + H]$^+$**	0.30	0.25	0.33	-0.16	0.12
**[LPC 19:0 + Na]$^+$**	0.37	0.45	0.33	0.21	-0.13
**[Lys-^{15}N + H]$^+$**	0.48	0.60	0.65	0.26	0.35
**[Lys-^{15}N + Na]$^+$**	0.28	0.67	0.51	1.39	0.81
**[Met-^{15}N + H]$^+$**	0.15	0.09	0.17	-0.38	0.14
**[Met-^{15}N + Na]$^+$**	0.24	0.37	0.27	0.52	0.12
**[PC 11:0/11:0 + H]$^+$**	0.64	0.58	0.72	-0.08	0.13
**[PC 11:0/11:0 + Na]$^+$**	0.76	1.15	0.77	0.51	0.01
**[PE 30:0 + H]$^+$**	3.01	2.09	2.50	-0.31	-0.17
**[PE 30:0 + Na]$^+$**	5.25	6.64	3.65	0.26	-0.30
**[PG 30:0 + H]$^+$**	-	6.35	3.68	-	-
**[PG 30:0 + Na]$^+$**	2.41	2.24	2.28	-0.07	-0.06
**[Phe-^{15}N + H]$^+$**	0.36	0.30	0.45	-0.17	0.25
**[Phe-^{15}N + Na]$^+$**	0.61	0.98	0.72	0.61	0.18
**[Pro-^{15}N + H]$^+$**	0.39	0.29	0.50	-0.25	0.28
**[Pro-^{15}N + Na]$^+$**	0.76	1.08	0.76	0.42	0.00
**[Ser-^{15}N + H]$^+$**	0.51	0.46	0.72	-0.11	0.40
**[Ser-^{15}N + Na]$^+$**	0.67	0.92	0.84	0.38	0.25
**[Thr-^{15}N + H]$^+$**	0.55	0.38	0.73	-0.30	0.34
**[Thr-^{15}N + Na]$^+$**	0.81	1.10	0.98	0.36	0.22
**[Trp-^{15}N + H]$^+$**	0.36	0.32	0.44	-0.12	0.21
**[Trp-^{15}N + Na]$^+$**	0.70	0.84	0.68	0.20	-0.03
**[Tyr-^{15}N + H]$^+$**	0.11	0.13	0.22	0.26	1.05
**[Tyr-^{15}N + Na]$^+$**	0.25	0.31	0.27	0.27	0.10
**[Val-^{15}N + H]$^+$**	0.66	0.49	0.78	-0.25	0.18
**[Val-^{15}N + Na]$^+$**	1.06	1.89	1.37	0.78	0.30
Average	0.89	1.18	1.09	0.21	0.28

Table S9. List of putative assignments of a methanolic rat brain extract analyzed with DIP-MS. Contains 218 features which corresponds to 120 metabolites when adducts are combined.

Putative annotations	Adduct	Theoretical m/z	Experimental m/z	Δ ppm
Phosphoglycerate	$[M + H]^+$	187.0002	187.0002	0.24
6-phosphogluconate	$[M + H]^+$	277.0319	277.0324	1.78
Acetylcholine	$[M]^+$	146.1176	146.1176	-0.21
Adenine	$[M + H]^+$	136.0618	136.0618	-0.35
Adenosine	$[M + H]^+$	268.1040	268.1042	0.93
	$[M + Na]^+$	290.0860	290.0849	-3.69
Adipic acid	$[M + K]^+$	185.0212	185.0209	-1.60
Ala	$[M + H]^+$	90.0550	90.0551	1.66
	$[M + Na]^+$	112.0369	112.0371	1.80
	$[M + K]^+$	128.0110	128.0110	-0.35
Arg	$[M + H]^+$	175.1190	175.1188	-0.98
Asn	$[M + H]^+$	133.0608	133.0607	-1.04
	$[M + Na]^+$	155.0428	155.0427	-0.83
	$[M + K]^+$	171.0167	171.0166	-0.31
Asp	$[M + H]^+$	134.0448	134.0447	-0.91
	$[M + Na]^+$	156.0268	156.0267	-0.82
	$[M + K]^+$	172.0008	172.0006	-0.93
C16 carnitine	$[M + H]^+$	400.3421	400.3421	-0.07
C18:2 carnitine	$[M + Na]^+$	446.3241	446.3262	4.78
C2 carnitine	$[M + H]^+$	204.1230	204.1230	-0.07
Carnitine	$[M + H]^+$	162.1125	162.1124	-0.69
	$[M + Na]^+$	184.0944	184.0944	-0.22
	$[M + K]^+$	200.0684	200.0682	-0.81
Carnosine	$[M + H]^+$	227.1139	227.1141	0.90
Cholesterol	$[M + H - H_2O]^+$	369.3516	369.3514	-0.49
Choline	$[M]^+$	104.1070	104.1073	2.66
Cit	$[M + H]^+$	176.1030	176.1028	-0.97
	$[M + K]^+$	214.0588	214.0587	-0.52
Citrate	$[M + K]^+$	230.9903	230.9904	0.61
c-LPA 18:2	$[M + H]^+$	417.2406	417.2401	-1.32
Creatine	$[M + H]^+$	132.0768	132.0767	-0.71
	$[M + Na]^+$	154.0588	154.0587	-0.89
	$[M + K]^+$	170.0328	170.0325	-1.67
Creatinine	$[M + H]^+$	114.0662	114.0664	1.51
	$[M + Na]^+$	136.0482	136.0480	-1.44
	$[M + K]^+$	152.0222	152.0221	-0.39
DHAP	$[M + H]^+$	171.0053	171.0054	0.50
Erythritol	$[M + K]^+$	161.0212	161.0211	-0.70
FA 18:1	$[M + K]^+$	321.2195	321.2192	-0.81
FA 20:4	$[M + Na]^+$	327.2300	327.2296	-1.18
	$[M + K]^+$	343.2039	343.2034	-1.41
FA 20:5	$[M + Na]^+$	325.2143	325.2141	-0.63
FA 22:4	$[M + K]^+$	371.2352	371.2346	-1.52
FA 22:6	$[M + Na]^+$	351.2294	351.2295	0.22
	$[M + K]^+$	367.2033	367.2035	0.60
GABA	$[M + H]^+$	104.0706	104.0709	2.96
	$[M + Na]^+$	126.0526	126.0526	-0.36
	$[M + K]^+$	142.0266	142.0266	0.19
Gln	$[M + H]^+$	147.0764	147.0764	0.21
	$[M + Na]^+$	169.0584	169.0583	-0.79
	$[M + K]^+$	185.0324	185.0323	-0.77
Glu	$[M + H]^+$	148.0604	148.0604	0.17
	$[M + Na]^+$	170.0424	170.0422	-1.01
	$[M + K]^+$	186.0164	186.0161	-1.40
Glucosamine	$[M + H]^+$	180.0866	180.0866	-0.04
Glucose	$[M + Na]^+$	203.0526	203.0523	-1.25
	$[M + K]^+$	219.0265	219.0267	0.75
Gly	$[M + H]^+$	76.0393	76.0393	0.54
	$[M + Na]^+$	98.0212	98.0215	3.25
	$[M + K]^+$	113.9953	113.9955	1.34
Glycerol phosphate	$[M + H]^+$	173.0210	173.0209	-0.33

Glycerol	[M + K]+	131.0105	131.0105	-0.13
Guanosine	[M + K]+	322.0549	322.0552	1.05
Caproic acid	[M + Na]+	139.0730	139.0728	-1.50
	[M + K]+	155.0470	155.0468	-1.25
His	[M + H]+	156.0768	156.0767	-0.46
	[M + Na]+	178.0587	178.0585	-0.85
	[M + K]+	194.0326	194.0326	-0.04
Homocarnosine	[M + H]+	241.1295	241.1297	0.95
Homocysteine	[M + K]+	173.9986	173.9988	1.12
Hydroxyproline	[M + Na]+	154.0475	154.0475	-0.32
Hypoxanthine	[M + H]+	137.0458	137.0457	-1.01
	[M + Na]+	159.0278	159.0277	-0.71
	[M + K]+	175.0018	175.0015	-1.44
Inosine	[M + H]+	269.0880	269.0883	1.07
	[M + Na]+	291.0700	291.0701	0.21
	[M + K]+	307.0440	307.0442	0.52
Lactate	[M + Na]+	113.0210	113.0211	0.98
	[M + K]+	128.9950	128.9949	-1.04
Leu	[M + H]+	132.1019	132.1018	-0.59
	[M + Na]+	154.0839	154.0838	-0.54
	[M + K]+	170.0578	170.0577	-0.36
LPC 16:0	[M + H]+	496.3403	496.3393	-2.09
	[M + Na]+	518.3222	518.3205	-3.29
	[M + K]+	534.2962	534.2949	-2.39
LPC 18:0	[M + K]+	562.3275	562.3271	-0.63
LPC 18:1	[M + H]+	522.3559	522.3548	-2.11
	[M + Na]+	544.3379	544.3365	-2.48
	[M + K]+	560.3118	560.3113	-0.93
LPC 20:0	[M + H]+	552.4029	552.4017	-2.11
LPC 20:4	[M + Na]+	566.3222	566.3218	-0.75
	[M + K]+	582.2962	582.2956	-1.04
LPC 22:6	[M + K]+	606.2962	606.2956	-1.00
LPE 18:0	[M + K]+	520.2805	520.2794	-2.08
LPE 20:4	[M + Na]+	524.2753	524.2740	-2.50
	[M + K]+	540.2492	540.2480	-2.25
LPE 22:6	[M + Na]+	548.2753	548.2738	-2.81
	[M + K]+	564.2492	564.2487	-0.85
Lys	[M + H]+	147.1128	147.1128	0.06
	[M + K]+	185.0688	185.0686	-1.02
Malate	[M + Na]+	157.0108	157.0107	-0.61
	[M + K]+	172.9848	172.9847	-0.69
Met	[M + H]+	150.0583	150.0583	0.23
	[M + Na]+	172.0403	172.0402	-0.72
	[M + K]+	188.0142	188.0143	0.60
monosodium glutamate	[M + Na]+	192.0242	192.0241	-0.75
N-acetylaspartate	[M + H]+	176.0553	176.0552	-0.53
	[M + Na]+	198.0373	198.0371	-1.16
	[M + K]+	214.0112	214.0111	-0.36
Neurine +	[M]+	86.0964	86.0965	1.39
Nicotinamide	[M + H]+	123.0553	123.0554	0.42
	[M + Na]+	145.0373	145.0372	-0.37
	[M + K]+	161.0113	161.0111	-1.33
Oxoproline	[M + H]+	130.0499	130.0499	-0.26
	[M + Na]+	152.0318	152.0318	-0.05
	[M + K]+	168.0058	168.0057	-0.83
Pantothenic acid	[M + K]+	258.0740	258.0741	0.49
PC 32:0	[M + H]+	734.5694	734.5692	-0.25
	[M + Na]+	756.5514	756.5509	-0.62
	[M + K]+	772.5253	772.5245	-0.99
PC 32:1	[M + H]+	732.5538	732.5542	0.54
	[M + Na]+	754.5357	754.5358	0.17
	[M + K]+	770.5097	770.5096	-0.07
PC 34:0	[M + H]+	762.6007	762.5992	-1.91
	[M + Na]+	784.5827	784.5794	-4.21
	[M + K]+	800.5566	800.5546	-2.47
PC 34:1	[M + H]+	760.5851	760.5847	-0.50

	$[M + Na]^+$	782.5670	782.5668	-0.25
	$[M + K]^+$	798.5410	798.5404	-0.78
PC O-34:1	$[M + H]^+$	746.6058	746.6047	-1.42
	$[M + Na]^+$	768.5878	768.5873	-0.60
	$[M + K]^+$	784.5617	784.5613	-0.46
PC 34:2	$[M + H]^+$	758.5694	758.5703	1.16
	$[M + Na]^+$	780.5514	780.5513	-0.09
	$[M + K]^+$	796.5253	796.5253	-0.04
PC 36:1	$[M + H]^+$	788.6164	788.6158	-0.74
	$[M + Na]^+$	810.5983	810.5985	0.22
	$[M + K]^+$	826.5723	826.5720	-0.36
PC 36:2	$[M + H]^+$	786.6007	786.6012	0.63
	$[M + Na]^+$	808.5827	808.5828	0.08
	$[M + K]^+$	824.5566	824.5569	0.35
PC 36:3	$[M + Na]^+$	806.5670	806.5687	2.14
PC 36:4	$[M + Na]^+$	804.5514	804.5508	-0.81
	$[M + K]^+$	820.5253	820.5255	0.18
PC 38:3	$[M + Na]^+$	834.5983	834.6011	3.40
PC 38:4	$[M + Na]^+$	832.5827	832.5822	-0.58
	$[M + K]^+$	848.5566	848.5566	0.05
PC 38:5	$[M + Na]^+$	830.5670	830.5667	-0.42
	$[M + K]^+$	846.5410	846.5416	0.67
PC 38:6	$[M + Na]^+$	828.5514	828.5507	-0.86
	$[M + K]^+$	844.5253	844.5251	-0.18
PC 40:4	$[M + K]^+$	876.5879	876.5873	-0.64
PC 40:6	$[M + Na]^+$	856.5827	856.5820	-0.85
	$[M + K]^+$	872.5566	872.5568	0.26
PC 40:7	$[M + Na]^+$	854.5670	854.5665	-0.55
	$[M + K]^+$	870.5410	870.5405	-0.61
PC 42:5	$[M + H]^+$	864.6477	864.6514	4.24
PC O-36:7	$[M + Na]^+$	784.5257	784.5255	-0.32
PC O-38:7	$[M + Na]^+$	812.5570	812.5569	-0.14
PC O-40:10	$[M + Na]^+$	834.5413	834.5412	-0.12
PC O-42:3	$[M + K]^+$	892.6561	892.6576	1.67
PE 36:1	$[M + Na]^+$	768.5519	768.5524	0.69
PE 38:3	$[M + Na]^+$	792.5519	792.5535	1.98
PE 38:4	$[M + Na]^+$	790.5362	790.5351	-1.40
	$[M + K]^+$	806.5102	806.5098	-0.46
PE 38:6	$[M + K]^+$	802.4789	802.4783	-0.78
PE 40:6	$[M + Na]^+$	814.5362	814.5349	-1.64
	$[M + K]^+$	830.5102	830.5098	-0.45
PE O-34:2	$[M + Na]^+$	724.5257	724.5261	0.50
	$[M + K]^+$	740.4996	740.4995	-0.12
PE O-36:2	$[M + Na]^+$	752.5570	752.5573	0.46
	$[M + K]^+$	768.5309	768.5305	-0.54
PE O-36:3	$[M + Na]^+$	750.5413	750.5407	-0.74
	$[M + K]^+$	766.5153	766.5150	-0.45
PE O-36:4	$[M + Na]^+$	748.5257	748.5273	2.11
PE O-36:5	$[M + K]^+$	762.4840	762.4835	-0.63
PE O-38:4	$[M + Na]^+$	776.5570	776.5588	2.37
PE O-38:5	$[M + K]^+$	790.5153	790.5147	-0.75
PE O-38:6	$[M + K]^+$	788.4996	788.4990	-0.81
PE O-38:7	$[M + K]^+$	786.4840	786.4837	-0.38
PE O-40:5	$[M + K]^+$	818.5466	818.5463	-0.34
PE O-40:7	$[M + K]^+$	814.5153	814.5147	-0.73
PE O-40:8	$[M + K]^+$	812.4996	812.4992	-0.48
Phe	$[M + H]^+$	166.0863	166.0862	-0.76
	$[M + Na]^+$	188.0683	188.0680	-1.51
	$[M + K]^+$	204.0423	204.0423	-0.24
Phosphocholine +	$[M]^+$	184.0733	184.0733	-0.07
Pro	$[M + H]^+$	116.0706	116.0708	1.34
	$[M + Na]^+$	138.0526	138.0524	-1.35
	$[M + K]^+$	154.0266	154.0266	-0.12
Ribose	$[M + Na]^+$	173.0421	173.0419	-0.83
Ser	$[M + H]^+$	106.0499	106.0501	2.19

	$[M + Na]^+$	128.0319	128.0318	-0.55
	$[M + K]^+$	144.0059	144.0059	-0.07
SM 18:0	$[M + H]^+$	731.6067	731.6054	-1.72
	$[M + Na]^+$	753.5887	753.5881	-0.83
	$[M + K]^+$	769.5626	769.5605	-2.75
Sodium Phosphate	$[M + Na]^+$	142.9482	142.9481	-0.75
Sorbitol	$[M + Na]^+$	205.0683	205.0683	0.22
Succinate	$[M + Na]^+$	141.0159	141.0156	-1.90
	$[M + K]^+$	156.9899	156.9898	-0.40
Taurine	$[M + H]^+$	126.0219	126.0221	1.55
	$[M + Na]^+$	148.0039	148.0039	-0.11
	$[M + K]^+$	163.9779	163.9778	-0.43
Thr	$[M + H]^+$	120.0655	120.0656	1.07
	$[M + Na]^+$	142.0475	142.0475	0.22
	$[M + K]^+$	158.0215	158.0215	-0.10
Trp	$[M + H]^+$	205.0972	205.0971	-0.57
Tyr	$[M + H]^+$	182.0812	182.0810	-0.88
	$[M + Na]^+$	204.0632	204.0631	-0.59
	$[M + K]^+$	220.0371	220.0373	0.87
Urea	$[M + Na]^+$	83.0216	83.0216	0.54
	$[M + K]^+$	98.9955	98.9958	2.80
Val	$[M + H]^+$	118.0863	118.0864	0.71
	$[M + Na]^+$	140.0683	140.0680	-1.97
	$[M + K]^+$	156.0423	156.0421	-1.48
Xanthine	$[M + Na]^+$	175.0226	175.0226	-0.01
	$[M + K]^+$	190.9966	190.9965	-0.73

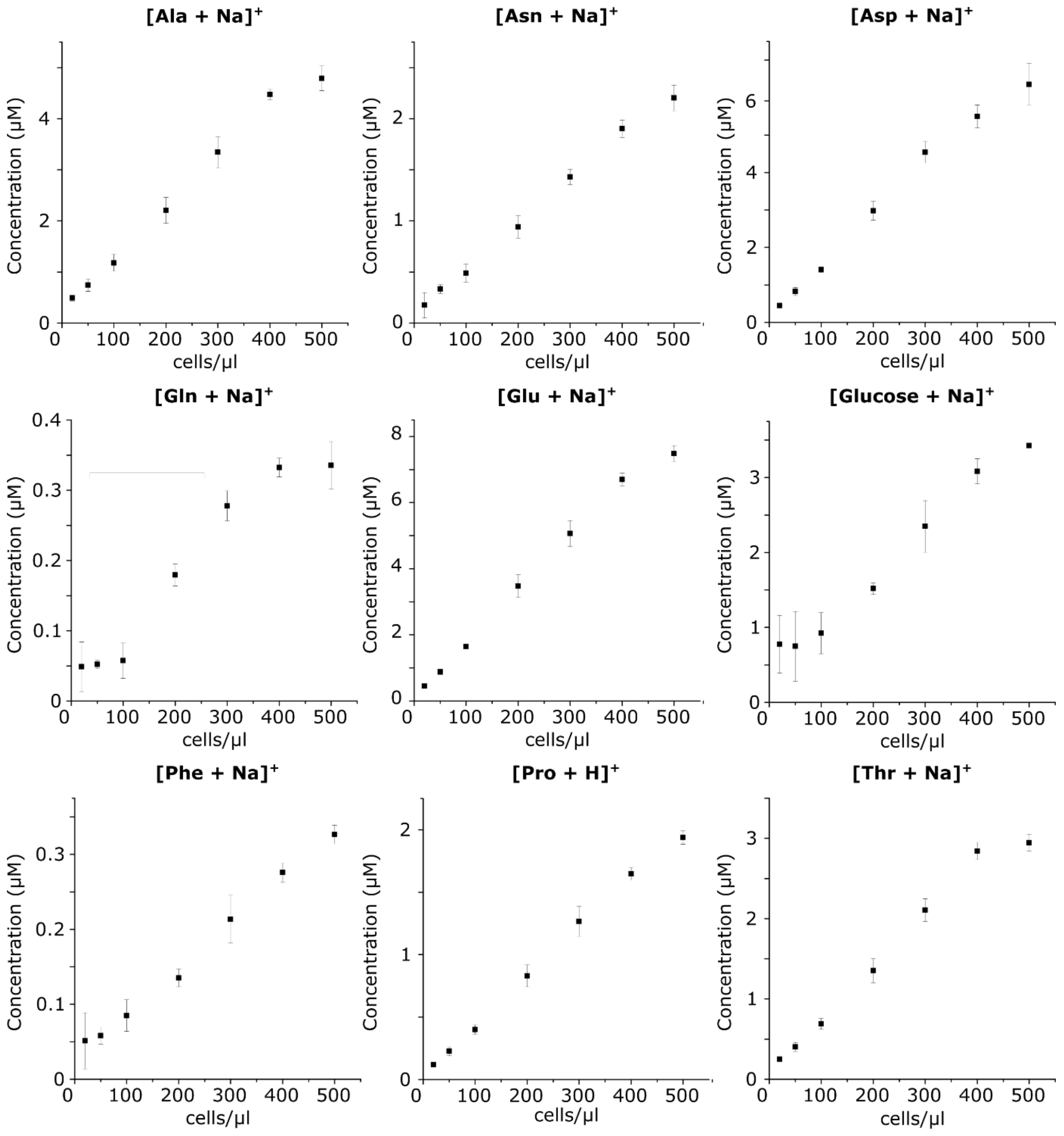

Figure S6. Individual plots of concentrations of endogenous small metabolites in 5 µl samples with cell densities ranging from 20 to 500 cells/µl. These compounds were quantified using the respective isotopically labelled internal standard.

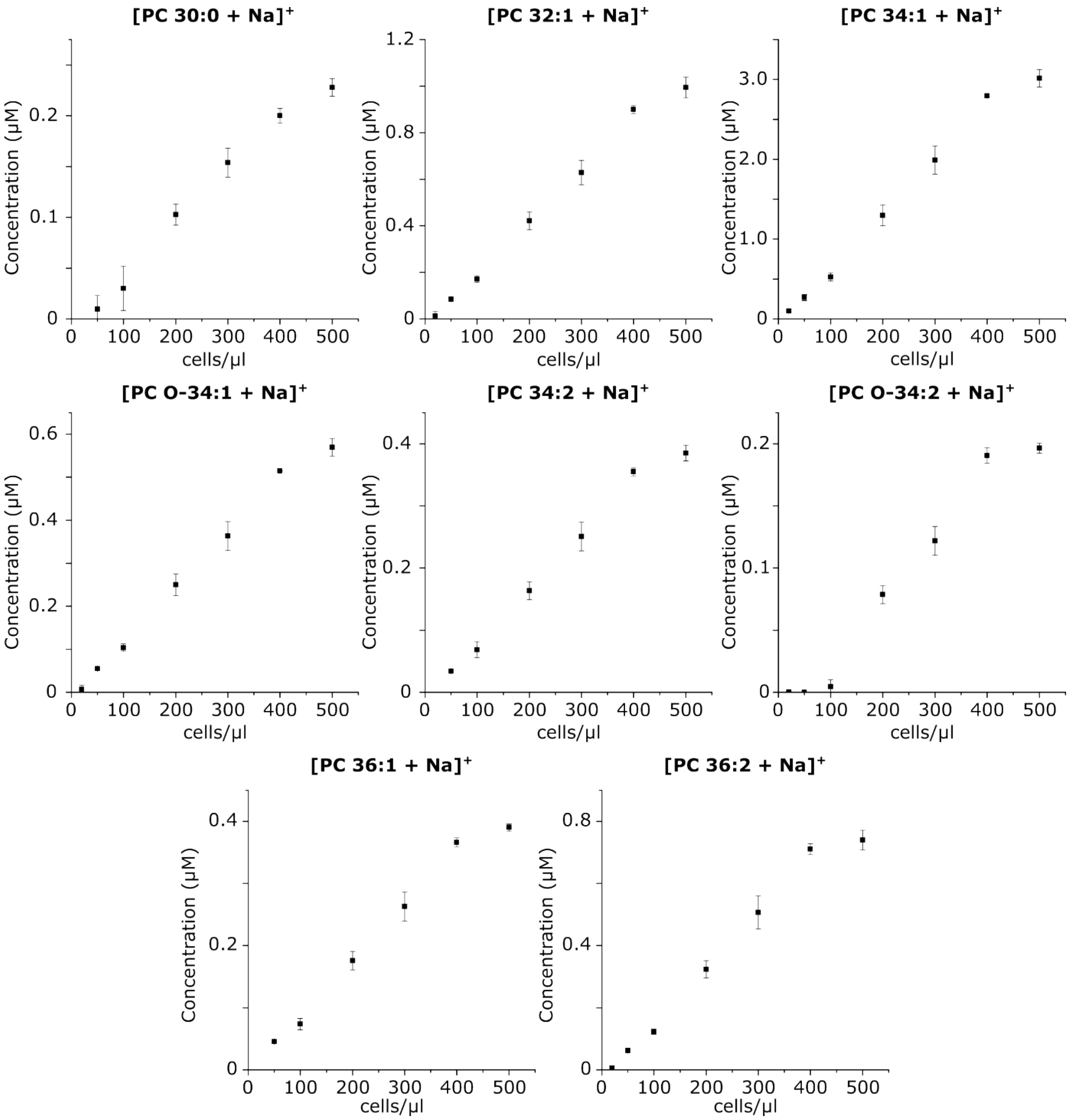

Figure S7. Individual plots of concentrations of endogenous lipids in 5 μl samples with cell densities ranging from 20 to 500 cells/μl. These compounds were quantified using the internal standard LPC 19:0.

Table S10. Significant features for non-target analysis present in the volcano plot of Figure 5. This data is the normalized with global median of the intensities of 191 putatively identified features.

	Fold change	log2(FC)	P-value
**[Sodium phosphate + H]$^+$**	0.37	-1.43	1.34E-16
**[Sorbitol + Na]$^+$**	13.9	3.79	1.49E-13
**[Sodium Phosphate + Na]$^+$**	0.33	-1.59	5.95E-12
**[Citrate + H]$^+$**	0.78	-0.36	1.10E-07
**[Asp + Na]$^+$**	0.41	-1.30	1.65E-07
**[Pro + Na]$^+$**	1.40	0.49	3.13E-07
**[Uracil + Na]$^+$**	0.76	-0.39	2.11E-06
**[Glucose + K]$^+$**	7.16	2.84	3.17E-06
**[Glucose + Na]$^+$**	7.51	2.91	6.94E-06
**[Hydroxyproline + Na]$^+$**	0.81	-0.30	2.50E-05
**[Choline]$^+$**	1.26	0.34	3.01E-05
**[C3 carnitine + H]$^+$**	0.70	-0.52	3.50E-05
**[acetylcarnitine + H]$^+$**	1.40	0.48	7.30E-05
**[Glycerylphosphorylethanolamine + Na]$^+$**	0.84	-0.25	7.94E-05
**[Gluconate + K]$^+$**	0.79	-0.34	1.80E-04
**[PE 38:5 + K]$^+$**	0.49	-1.04	1.95E-04
**[Gly + Na]$^+$**	0.78	-0.37	4.79E-04
**[Asn + Na]$^+$**	0.75	-0.42	4.95E-04
**[GABA + H]$^+$**	0.79	-0.34	6.89E-04
**[Val + Na]$^+$**	0.92	-0.13	0.001
**[Taurine + Na]$^+$**	0.84	-0.26	0.002
**[Inosine + Na]$^+$**	174.3	7.45	0.003
**[PC e36:7 + K]$^+$**	0.83	-0.27	0.004
**[FA 10:0 + Na]$^+$**	0.82	-0.29	0.005
**[Dimethylaniline H]$^+$**	0.81	-0.31	0.005
**[FA 12:0 + Na]$^+$**	0.79	-0.34	0.006
**[Ala + Na]$^+$**	1.10	0.14	0.006
**[Val + H]$^+$**	0.85	-0.23	0.007
**[Nonanoate + Na]$^+$**	0.82	-0.28	0.008
**[Adipic acid + Na]$^+$**	0.84	-0.25	0.008
**[FA 18:1 + Na]$^+$**	1.29	0.36	0.008
**[Octanoate + Na]$^+$**	0.82	-0.29	0.009
**[GABA + Na]$^+$**	0.87	-0.20	0.013
**[Arabitol + Na]$^+$**	3.22	1.69	0.017
**[Asp + H]$^+$**	0.01	-7.02	0.019
**[Glycerol K]$^+$**	0.72	-0.47	0.020
**[Gly + K]$^+$**	0.30	-1.73	0.021
**[Succinate + Na]$^+$**	0.82	-0.28	0.022
**[FA 16:0 + Na]$^+$**	0.87	-0.20	0.023
**[Thr + Na]$^+$**	0.59	-0.77	0.023
**[Lactate + Na]$^+$**	0.76	-0.40	0.024
**[Caproic acid + Na]$^+$**	0.84	-0.26	0.025
**[Taurine + H]$^+$**	0.02	-6.09	0.025
**[Creatine + H]$^+$**	0.91	-0.13	0.026
**[Glycerol + Na]$^+$**	0.85	-0.24	0.027
**[Creatinine + Na]$^+$**	0.95	-0.07	0.028
**[Ser + Na]$^+$**	0.87	-0.20	0.029
**[PC 28:0 + Na]$^+$**	4.07	2.02	0.032
**[PC 32:0 + Na]$^+$**	1.71	0.77	0.034
**[Taurine + K]$^+$**	0.74	-0.43	0.035
**[Putrescine + H]$^+$**	0.80	-0.32	0.038
**[FA 12:0 + K]$^+$**	0.85	-0.23	0.039
**[Lactate + K]$^+$**	0.66	-0.59	0.039
**[Urea + K]$^+$**	0.31	-1.67	0.042
**[Cer d42:2 + K]$^+$**	0.46	-1.13	0.046
**[Glu + H]$^+$**	0.84	-0.25	0.046

Table S11. Top pathways identified with MetaboAnalyst Network tool considering significant features from non-targeted analysis.

Pathway	Hits	P-value
Alanine, aspartate and glutamate metabolism	7	2.06E-06
Glycine, serine and threonine metabolism	6	4.93E-05
Arginine and proline metabolism	6	1.71E-04
Glyoxylate and dicarboxylate metabolism	5	6.61E-04
Fatty acid biosynthesis	3	0.001
Arginine biosynthesis	3	0.004
Butanoate metabolism	3	0.005
Citrate cycle (TCA cycle)	3	0.006
Pantothenate and CoA biosynthesis	3	0.007
Glutathione metabolism	3	0.010
Valine, leucine and isoleucine biosynthesis	2	0.015
Glycerophospholipid metabolism	2	0.038

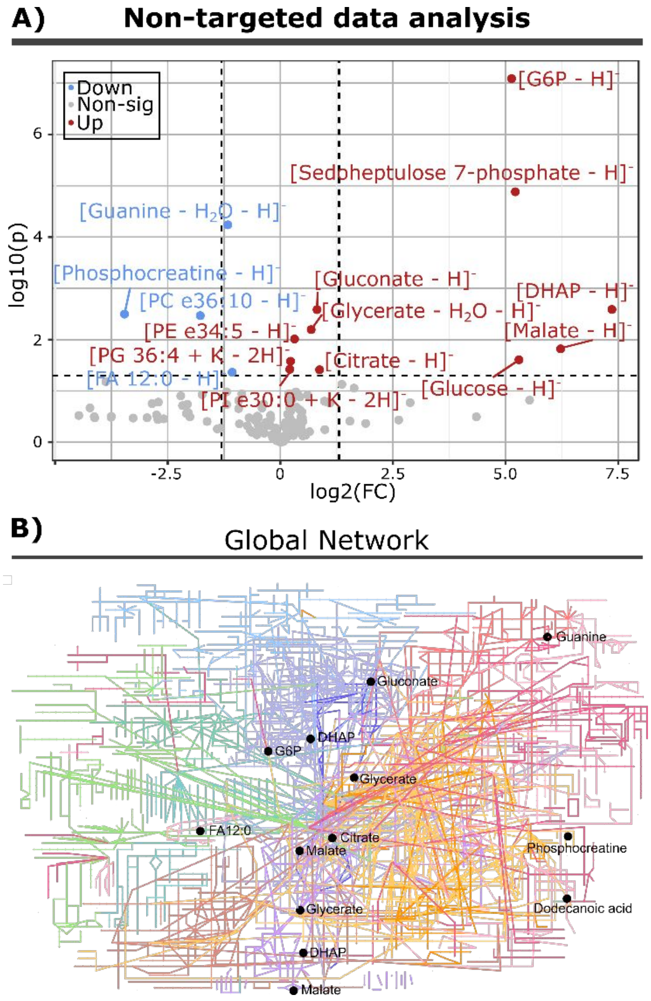

Figure S8. Non-targeted metabolite profiling of INS-1 cells exposed to either low (1 mM) or high (20 mM) glucose of data collected in negative mode. A) Non-targeted data analysis: Volcano plot originated from the global median normalization of a total of 176 features putatively assigned. A total of 15 metabolites were considered significant ($p < 0.05$) according to Student's t-test analysis and fold change higher than 1.04. Red and blue correspond to up and down regulated features, respectively, and in grey are the non-significant features. B) Network: The global metabolomic network highlighting in black dots some of the significantly altered metabolites.

Table S12. Significant features for non-target analysis of data collected in negative mode present in the volcano plot of Figure S8. This data is the normalized with global median of the intensities of 176 putatively identified features.

	Fold change	log2(FC)	P-value
[G6P - H]$^-$	35.10	5.133	8.19E-08
[Sedoheptulose 7-phosphate - H]$^-$	37.17	5.216	1.31E-05
[Guanine	0.446	-1.166	5.75E-05
[DHAP - H]$^-$	163.6	7.354	0.003
[Gluconate - H]$^-$	1.759	0.814	0.003
[Phosphocreatine - H]$^-$	0.091	-3.459	0.003
[PC e36:11 - H]$^-$	0.292	-1.778	0.003
[Glycerate	1.608	0.685	0.006
[PE e34:6 - H]$^-$	1.245	0.316	0.010
[Malate - H]$^-$	74.72	6.223	0.015
[Glucose - H]$^-$	39.28	5.296	0.025
[PG a 36:4 - H]$^-$	1.170	0.226	0.027
[PI e 30:0 - H]$^-$	1.155	0.208	0.038
[Citrate - H]$^-$	1.828	0.870	0.039
[FA 12:0 - H]$^-$	0.476	-1.070	0.043

Table S13. Top pathways identified with MetaboAnalyst Network tool considering significant features from non-targeted analysis of data collected in negative mode.

Pathway	**Hits**	**P-value**
Glycerolipid metabolism	2	8.54E-04
Glyoxylate and dicarboxylate metabolism	3	8.99E-04
Citrate cycle (TCA cycle)	2	0.005
Inositol phosphate metabolism	2	0.008
Pentose phosphate pathway	2	0.009
Neomycin, kanamycin and gentamicin biosynthesis	1	0.014
Fatty acid biosynthesis	1	0.066
Starch and sucrose metabolism	1	0.086
Glycerophospholipid metabolism	1	0.086
Pyruvate metabolism	1	0.123
Fructose and mannose metabolism	1	0.129
Alanine, aspartate and glutamate metabolism	1	0.176
Glycine, serine and threonine metabolism	1	0.188
Arginine and proline metabolism	1	0.227
Purine metabolism	1	0.366
